# User perceptions of RBI-approved P2P digital lending apps: an NLP, machine learning, and deep learning approach

**DOI:** 10.3389/frai.2025.1708080

**Published:** 2026-01-12

**Authors:** Kunchakara Raja Sekhar, Shaiku Shahida Saheb

**Affiliations:** VIT-AP School of Business, VIT-AP University, Amaravati, AP, India

**Keywords:** digital lending, RBI-approved apps, fintech, user perception, sentiment analysis

## Abstract

**Introduction:**

Digital lending, also known as alternative lending, refers to fintech platforms that offer quick and easy loans through digital channels, bypassing many of the limitations of traditional banking. Since the mid-2000s, digital lending has become a major fintech innovation, with rapid growth in India driven by financial inclusion measures. However, the sector continues to face challenges, including fraud, transparency issues, and consumer dissatisfaction. The primary objective of this study was to understand how consumers perceive and assess India’s RBI-approved P2P digital lending apps by analyzing a large dataset of customer feedback to identify strengths, weaknesses, and overall satisfaction levels.

**Methods:**

The study analyzed a final dataset of 15,408 user reviews collected from seven RBI-approved digital lending platforms: 5Paisa, Faircent, i2iFunding, LenDenClub, CashKumar, Lendbox, and IndiaMoneyMart derived from an initial 15,537 reviews. The cleaned data was then examined using natural language processing, topic modeling, and supervised machine learning and deep learning models to identify key themes and evaluate predictive performance.

**Results:**

Topic modeling identified 11 recurring topics. Sentiment analysis revealed that 55% of evaluations were positive, 41% were negative, and 4% were neutral. Strengths included loan disbursement, withdrawals, and EMI payments, while weaknesses involved interface design, transparency around rejections, and login functionality. Comparative data revealed that IndiaMoneyMart and i2iFunding received the highest user satisfaction, while 5Paisa and Lendbox trailed due to recurring complaints about transparency, accessibility, and overall user experience. In terms of modeling, the deep learning model VGG16 and ensemble machine learning techniques (XGBoost, CatBoost, and LightGBM) consistently achieved the highest predictive accuracy (up to 0.88), outperforming simpler models such as decision trees and ResNets.

**Discussion:**

The findings indicate that digital lending platforms support financial inclusion but require improvements in user interface and user experience, better transparency in loan decisions, and stronger customer support. Addressing these areas can help strengthen trust and promote long term adoption of digital lending services.

## 1 Introduction

Digital lending, also known as alternative lending, provides affordable, easily accessible loans through online platforms. Zopa, the first such platform, debuted in the UK in March 2005 (Pang et al., 2022). In the early 2010s, digital lending gained popularity alongside the explosive expansion of fintech. Between 2012 and 2020, digital lending platforms experienced significant growth, offering additional credit access options (Cevik, 2024). During this period, sophisticated financial services powered by technology emerged, swiftly revolutionizing the conventional banking system (Anifa et al., 2022). Currently, numerous platforms offer commercial lending services, such as Easy Credit, PPDai, Lending Club, Zopa, and Prosper (Chen et al., 2014). The digital lending industry has expanded considerably over the past few years (Sarungu, 2020). It is regarded as one of the most transformative fintech innovations, reshaping traditional banking and increasing access to credit (Modi and Kesarani, 2023). This rapid growth has been driven by the adoption of new technology and shifting customer demands, making borrowing and lending faster, easier, simpler, and more convenient through digital channels (Modi and Kesarani, 2023). Moreover, fintech, philanthropy, development, and the monetization of digital footprints drive the expansion of digital lending (Gabor and Brooks, 2017). These lending platforms significantly improve financial inclusion and access to capital, particularly in emerging nations (Zetzsche et al., 2017). In countries such as India, fintech has transformed the way consumers engage with financial institutions. Since 2014, India’s digital financial industry has witnessed rapid growth. Government-led initiatives such as demonetization have encouraged people to shift from cash to digital transactions (Ghosh and Hom Chaudhury, 2022). Furthermore, the implementation of the Pradhan Mantri Jan Dhan Yojana in August 2014 has significantly improved financial inclusion by simplifying the process of opening bank deposit accounts (Barik and Sharma, 2019).

However, fraud and identity theft remain significant challenges in digital lending, as dishonest actors exploit personal data for illicit activities and financial gain (Saunders and Zucker, 1999). These crimes have increased due to the expansion of internet services and digital transactions, particularly during and after the COVID-19 pandemic (Luong and Ngo, 2024). To mitigate these risks, additional security measures such as multi-factor authentication, secure payment gateways, and encryption are implemented (Ogunola et al., 2024). In India, the Reserve Bank of India (RBI) regulates digital lending platforms through its 2017 Master Directions (Khan et al., 2024), under which Faircent.com emerged as the country’s first peer-to-peer lending platform (Khatri, 2019). As fintech continues to evolve, technologies such as blockchain and smart contracts have become key tools for enabling secure, transparent, and sustainable digital financing solutions (Elias et al., 2024). It enhances transaction security, transparency, and trust, while smart contracts enable automated, secure enforcement of lending arrangements (Omowole et al., 2024). With loan origination, repayment, and collateral management, distributed ledger systems ensure immutable, transparent transactions, reducing fraud and enhancing trust (Rijanto, 2021). These technologies are particularly useful in the context of green finance and microfinance, where accountability and transparency are vital (Elias et al., 2024; Omowole et al., 2024). To handle the increasing complexity of lending applications and risks associated with P2P platforms, policymakers are using machine learning technologies to process vast amounts of data to inform regulatory and financial decisions (Xu et al., 2021). These technologies improve the security and efficiency of lending processes while also promoting environmental sustainability by incorporating ecological considerations into financial decision-making (Addy et al., 2024).

## 2 Literature review

The banking industry has been revolutionized by digital lending, which uses technology to streamline loan processes and increase credit availability (Mallinguh and Wasike, 2025). In recent years, the digital lending industry has grown rapidly (Sarungu, 2020). The growing popularity of online loans each year is driven by the expansion of the Internet and the rise of big data (Liu, 2025). Several commercial lending platforms have been made available, including Prosper, PPDai, Lending Club, Zopa, and Easy Credit (Chen et al., 2014). As per the Reserve Bank of India (RBI), 27 NBFC-P2P platforms have been registered under the 2023 Directions. These revolutionary force in the fintech industry, competing with traditional lending strategies. The integration of advanced technologies such as Big Data, AI, and Machine Learning (ML) has revolutionized credit assessment and risk management processes (Aldboush and Ferdous, 2023; Bazarbash, 2019). These technologies empower fintech firms to analyze vast datasets, including non-traditional sources (Bazarbash, 2019; Warin and Stojkov, 2021).

Digital lending apps have become extremely popular in recent years. Studies are now applying machine learning to study customer reviews and opinions, with these models helping to sort and interpret user feedback more effectively (Alawaji, 2025). While positive views emphasize its role in financial inclusion and efficiency, negative perspectives focus on ongoing challenges of risk, regulation, and disclosure (Maulida and Surbakti, 2024). However, borrowers’ perceived trust had a small impact on the incentive to use the P2P lending network (Gupta and Mahajan, 2023). According to reviews, borrowers were largely delighted with the platform’s services and speedy loan processing, but convenience of use, cost, and risk were less important. Both lenders and borrowers experienced problems (Gupta and Mahajan, 2023). Meanwhile, the study helps borrowers and lenders select appropriate applications and enables P2P platforms to assess their strengths and weaknesses. The study focuses on user-generated information, particularly online reviews, to examine which service features people evaluate and how these characteristics predict a consumer’s recommendation (Siering et al., 2018).

To achieve greater predictive power and flexibility, interpretable ML models autonomously identify internal structure and correlations, thereby challenging conventional statistical methods (Huang et al., 2004). Big data risk management systems have progressed from the fundamentals of machine learning approaches to more advanced deep learning techniques (Bao et al., 2024). Among the models used to predict loan default, logistic regression is among the most frequently used to estimate the probability of successful loan funding on peer-to-peer lending platforms (Puro et al., 2010). Another highly regarded method is the Support Vector Machine (SVM), a sophisticated machine learning technique based on statistical learning theory, known for delivering consistently high performance across a wide range of applications (Huang et al., 2004). Among classification and prediction tools in machine learning, decision trees are widely used for their simplicity and effectiveness. Studies have shown that machine learning models can effectively evaluate personal credit information and predict the likelihood of loan default. Of these models, the Deep Neural Network achieved the best accuracy of 0.94 (Liu, 2025). Similarly, a study using Naïve Bayes algorithms achieved 94% accuracy and identified several key factors influencing loan success, including interest rate, repayment time, loan description, credit grade, loan history, gender, and credit score (Vedala and Kumar, 2012). Meanwhile, another study employed multivariate logistic regression to predict both prepayment and default risks, two critical events associated with loan termination and creditor profit loss, achieving an overall model accuracy of 76.63%. Additionally, a separate study found that using LightGBM to forecast default risk on digital lending platforms could enhance lending clubs’ revenues, achieving a prediction accuracy of 68% (Ko et al., 2022).

The application of ML algorithms, including neural networks and ensemble models, has significantly improved the accuracy and efficiency of financial decision-making (Odei-appiah and Adjei, 2021). However, the widespread adoption of these advanced technologies also introduces several ethical, social, and regulatory challenges. These concerns include algorithmic bias, discrimination, lack of transparency, and potential violations of data privacy (Aldboush and Ferdous, 2023). Issues could result in digital financial exclusion, particularly through algorithmic redlining, in which automated systems deny credit based on proxy variables that correlate with race, income, or geographic location (Bazarbash, 2019). Additionally, the expansion of digital lending into riskier, less-regulated segments of the financial system has posed ongoing challenges for regulators and policymakers. Previous studies on P2P lending and crowdfunding have also explored how user sentiment and comments affect funding performance, interest rates, and default probabilities. According to certain studies, the default likelihood and cost of capital are only adversely impacted by favorable improvements in media and social media for P2P lending platforms (Wang et al., 2020). In conclusion, although digital lending powered by AI and ML offers substantial promise to enhance access to credit and operational efficiency, it also requires careful consideration of ethical, legal, and systemic risks. To ensure responsible innovation, maintain financial stability, and safeguard consumer rights, the development of robust regulatory frameworks and effective oversight mechanisms has been emphasized (Cevik, 2024) (Table 1).

**Table 1:** Model comparison with earlier research.

Title of the study	Author/year	Methodology	Finding
Lender Trust on P2P Lending: Analysis Based on Sentiment Analysis of Comment Text	Niu et al. (2020)	The paper employs a lexicon-based sentiment analysis method to assess lenders’ trust. BERT – validation and comparison. LDA-analyze key text topics.	A study found that, with an accuracy of 97%, lenders generally have a positive attitude toward peer-to-peer lending; however, this opinion diminishes over time. Their key worries are yield, security, and compliance, with negative sentiments primarily focused on security and regulatory compliance.
Customer satisfaction in peer-to-peer lending platforms: A text mining and sentiment analysis approach	Kumari et al. (2025)	The study employed a quantitative text analytics technique to identify thematic clusters from user evaluations using the CONCOR (Convergence of Iterated Correlations) method.	According to the results, user experience, customer service, and borrowing options are the main factors influencing customer satisfaction on P2P lending platforms. The sentiment model achieved an F1-score of 0.81, while regression analysis explained over 52% of the variance in satisfaction (*R*^2^ = 0.527), confirming the reliability of these behavioral insights.
Borrower Sentiment on P2P Lending in Indonesia Based on Google Play Store Reviews	Pohan et al. (2020)	The study investigated user attitudes toward Indonesian P2P lending sites using Naïve Bayes and random forest classification methods.	The study reveals, with 98% accuracy, that Indonesian P2P lenders prioritize loan approval and disbursement speed over platform security, with the majority of reviews focusing on loan processing speed.
Public Perception of Online P2P Lending Applications	Khan et al. (2024)	Three feature extraction methods were used in the study: hashing, TF-IDF, and Bag-of-Words (BOW). The VADER sentiment analysis approach was used. We employed a variety of machine learning models, including random forests, SVMs, decision trees, XGBoost, logistic regression, and KNN.	With an accuracy of 94% considering the results of the study, overall, Lendbox had the best default rate and user interface, LenDenClub had the most positive ratings, and i2i Funding had the best document verification. These results point to areas where P2P platforms, lenders, and borrowers can all benefit.
Analysis of Google Play Store’s Sentiment Review on Indonesia’s P2P Fintech Platform	Amrie et al. (2022)	The study employed text preprocessing and TF-IDF, used Data Miner for web scraping, and used Naïve Bayes to classify sentiments.	According to 77% of positive reviews, the majority of users had positive opinions of Indonesian P2P lending apps, mostly praising their quick loan approval and simple application processes. Poor communication, delayed payouts, and data privacy concerns were the main topics of negative reviews. Overall, the results indicate that increasing service effectiveness, security, and openness can boost customer satisfaction and confidence.
Posts and reviews in P2P online lending platforms: a sentiment analysis and cross-cultural comparison	Wang et al. (2022)	To investigate the relationship between borrower communication behavior and loan funding performance, the study employed logistic regression.	The study reports regression results (*R*^2^ = 0.286; *β* = 0.223, *p* < 0.01) showing that user-generated content, including borrower posts and reviews, serves as a key trust signal that enhances funding likelihood and reduces information asymmetry in P2P lending.
Proposed model	Feature extraction- BOW, TF-IDF, Hashing, word2Vec, FastText, BERT, GLloVe. Algorithms ML- random forest, SVM, decision tree, logistic regression, XGBoost, CatBoost, AdaBoost, LightGBM. DL- VGC16, BiLSTM, ResNet.		The investigation found that, while RBI-approved P2P lending applications are widely regarded and valued for their quick loan and repayment services, user satisfaction varies. With an accuracy of 88% India Money Mart and i2iFunding are the most reliable apps, while others struggle with interface design, rejections, and communication breakdowns. On the modeling front, ensemble ML and VGG16 (deep learning) outperform, demonstrating that AI-powered text analytics can accurately capture consumer perceptions in digital lending.

Previous research on peer-to-peer lending and crowdfunding has examined how user comments and sentiments affect factors such as fundraising success, interest rates, and default rates (Gupta and Mahajan, 2023). Khan et al. (2024) analyzed Google Play Store reviews to assess user perceptions of P2P lending systems. They found that customers prioritize speedy loan approvals, transparency, and responsive services as the key drivers of satisfaction. The majority of earlier P2P lending research was platform-specific, employed lexicon-based or simple machine learning techniques, and neither integrated deep learning nor conducted extensive comparisons across regulated apps (Niu et al., 2020). Text mining and sentiment analysis have also been utilized in several studies to explore how users interact with financial technologies. A text mining analysis revealed that reliability, usability, and security are the most critical factors in determining user satisfaction with P2P payment services. This strategy aligns with the current study’s emphasis on eliciting user perceptions to better understand how Indians perceive digital lending apps (Perea-Khalifi et al., 2024). There aren’t many studies that connect sentiment findings to behavioral theories, such as trust-risk frameworks or TAM. This leaves a research void for a thorough, theory-driven investigation that assesses user attitudes across several P2P lending platforms regulated by the RBI, utilizing topic modeling and sophisticated ML-DL models.

This study is the first to use extensive user-generated data to compare seven P2P lending apps in India that are regulated by the RBI. It combines hybrid ML–DL models, topic modeling, and sophisticated embeddings to increase sentiment prediction accuracy and offer empirical insights into satisfaction, usability, and trust. This study demonstrates how perceived simplicity, utility, and transparency impact the adoption of digital lending by integrating data-driven analysis with TAM, UTAUT, and trust-risk theories. This sets a standard for future fintech sentiment research and provides useful advice for improving platform performance and user trust.

### 2.1 Theoretical foundation

Theoretical frameworks such as the Technology Acceptance Model (TAM), the Unified Theory of Acceptance and Use of Technology (UTAUT), and Consumer Decision-Making Process theories (Chen and Zhou, 2016), along with the SERVQUAL Model and Trust Risk Theory, have been widely used to explain the adoption of digital financial services and lending platforms (Abu-taieh et al., 2022). While these models effectively describe user acceptance of new technologies, they often overlook post-adoption outcomes, such as borrower satisfaction, changes in financial behavior, and sustainable credit practices, which would provide stronger theoretical support (Singh, 2020). There is growing recognition of the need for studies that prioritize borrowers and examine the broader socio-economic and psychological impacts of digital borrowing, which remain underexplored in culturally diverse and economically stratified contexts (Wang et al., 2020). The Technology Acceptance Model (TAM) was proposed by Davis (1989) to explain how users adopt and continue using FinTech applications. It suggests that technology adoption is primarily influenced by two perceptions: perceived usefulness and perceived ease of use (Sylvie and Pascal, 2021). Similarly, the UTAUT framework is applied to interpret users’ sentiments toward FinTech apps, in which constructs such as performance expectancy and effort expectancy are reflected in users’ perceptions of app usefulness and ease of use (Venkatesh et al., 2019). In this context, trust and satisfaction further influence users’ behavioral intentions to continue using the platform. The SERVQUAL Model extends this understanding by emphasizing that service quality drives satisfaction and loyalty across five dimensions: reliability, responsiveness, assurance, empathy, and tangibles (Jain and Gupta, 2004). In online lending, users’ willingness to engage also depends on perceived trust, security, and reduced uncertainty, as explained by Trust-Risk theory. Prior studies have employed TAM to explain consumer behavior and their propensity to embrace technological improvements (Baron et al., 2006). In the context of digital lending and FinTech apps, these constructs expanded to include trust and transparency, which play a critical role in shaping user confidence and satisfaction. Users who perceive a platform as transparent and reliable are more likely to trust it, leading to greater satisfaction and continued use (Yadav, 2024). Thus, these theories collectively provide a strong foundation for linking user perceptions, trust, and satisfaction with technology adoption behavior observed in online reviews. This framework guides the interpretation of sentiment analysis results and explains how positive user experiences translate into greater acceptance of FinTech services (Table 2).

**Table 2:** Mapping of P2P App Review Topics to Theoretical Constructs.

S. no.	Variable/review topic	Underlying construct	Supporting theory/framework
1	App interface	Perceived Ease of Use/System Quality	Technology Acceptance Model (TAM): Is a Success Model
2	Application experience	Perceived Ease of Use/User Satisfaction	TAM; Expectation Confirmation Theory (ECT)
3	CIBIL and credit score	Perceived Trust/Information Quality	Trust Risk Theory; Is Success Model
4	Customer service	Service Quality (Responsiveness, Empathy)	SERVQUAL Model
5	Document verification	Perceived Reliability/Trust/Service Quality	SERVQUAL; Trust Risk Theory
6	Loan process	Performance Expectancy/Reliability	UTAUT; SERVQUAL
7	Loan rejection	Perceived Risk/Disconfirmation	Trust Risk Theory; ECT
8	Login issues	Effort Expectancy/System Quality	UTAUT; IS Success Model
9	OTP and verification	Facilitating Conditions/Security Assurance	UTAUT; Trust Risk Theory
10	Repayment and EMI	Assurance/Perceived Usefulness/Trust	SERVQUAL; TAM; Trust Risk Theory
11	Withdrawal	Performance Expectancy/Reliability	UTAUT; SERVQUAL

Although this study employs machine learning and deep learning models for sentiment analysis, the theoretical interpretation is grounded in the UTAUT model, which highlights how factors such as trust, transparency, and perceived ease of use influence users’ intentions to adopt FinTech applications.

## 3 Objectives and methodology

### 3.1 Objectives

**Figure 1:**
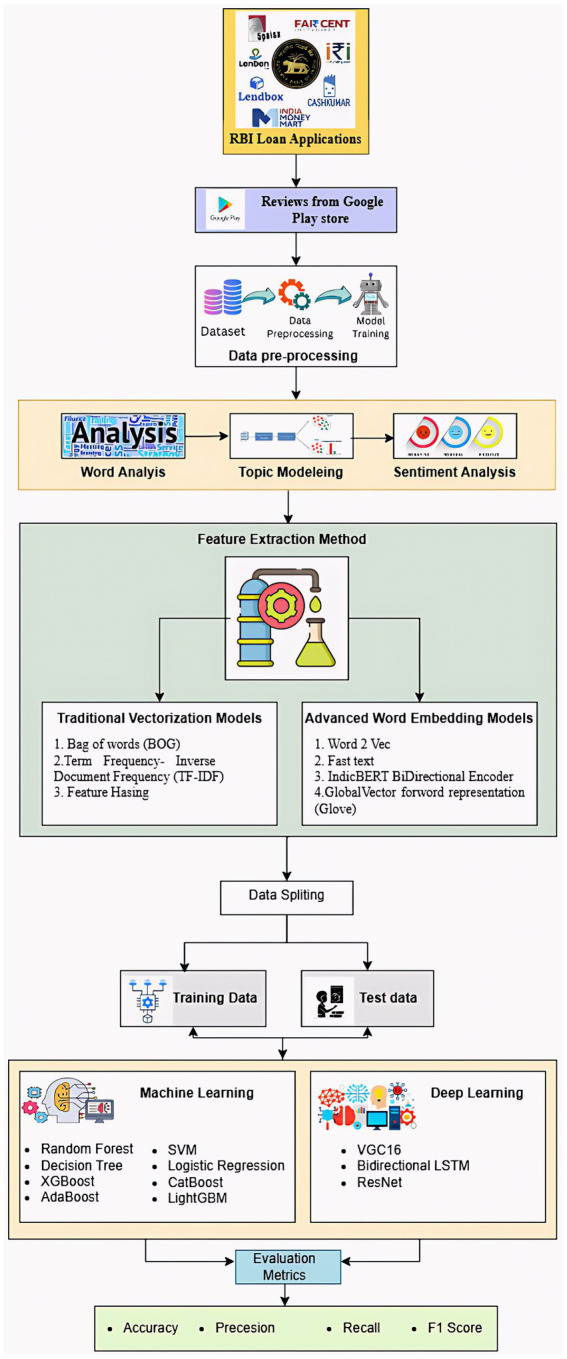
Proposed methodology.

1. To analyze user-generated reviews of regulated P2P lending applications in India using text mining and sentiment analysis.

2. To conduct a comparative analysis of digital lending applications by applying machine and deep learning models, aiming to evaluate app performance, borrower satisfaction, and trustworthiness.

3. To provide a theory-driven interpretation of user attitudes and behavioral intentions toward digital lending platforms (Figure 1).

### 3.2 Materials and methods

This study employs machine learning (ML), deep learning (DL), and natural language processing (NLP) techniques to analyze user feedback from digital lending apps approved by the RBI. Seven popular peer-to-peer lending platforms were examined using purposive selection between January 2020 and June 2024. The selection criteria included an active presence in the Google Play Store and an adequate volume of user reviews. Reviews were collected through a Python script built with the Google Play Scraper API. Extraction relied on specific application package IDs rather than keyword searches. The analyzed apps were 5Paisa (com.a5paisa.trade), Faircent (com.faircent.app), I2IFunding (com.i2ifunding.app), LendenClub (in.infra.lendenclub), CashKumar (com.cashkumar.loan), Lendbox (com.lendbox.app), and IndiaMoneyMart. These platforms were chosen for their accuracy in text mining and sentiment analysis, their relevance to the Indian digital lending sector, and their regulatory approval under the RBI’s NBFC-P2P framework. The collected fields included review ID, user name, score, review content, timestamp, and app metadata. Because the Google Play Store aggregates feedback across multiple releases, the dataset includes reviews from various app versions. Focusing on these platforms ensured data consistency and meaningful comparability between applications. A total of 15,537 reviews were scraped from the Google Play Store using a Python-based scraping technique. To prepare the raw textual data for analysis, preprocessing techniques such as noise reduction, lowercasing, tokenization, and stopword removal were applied.

**Figure 2:**
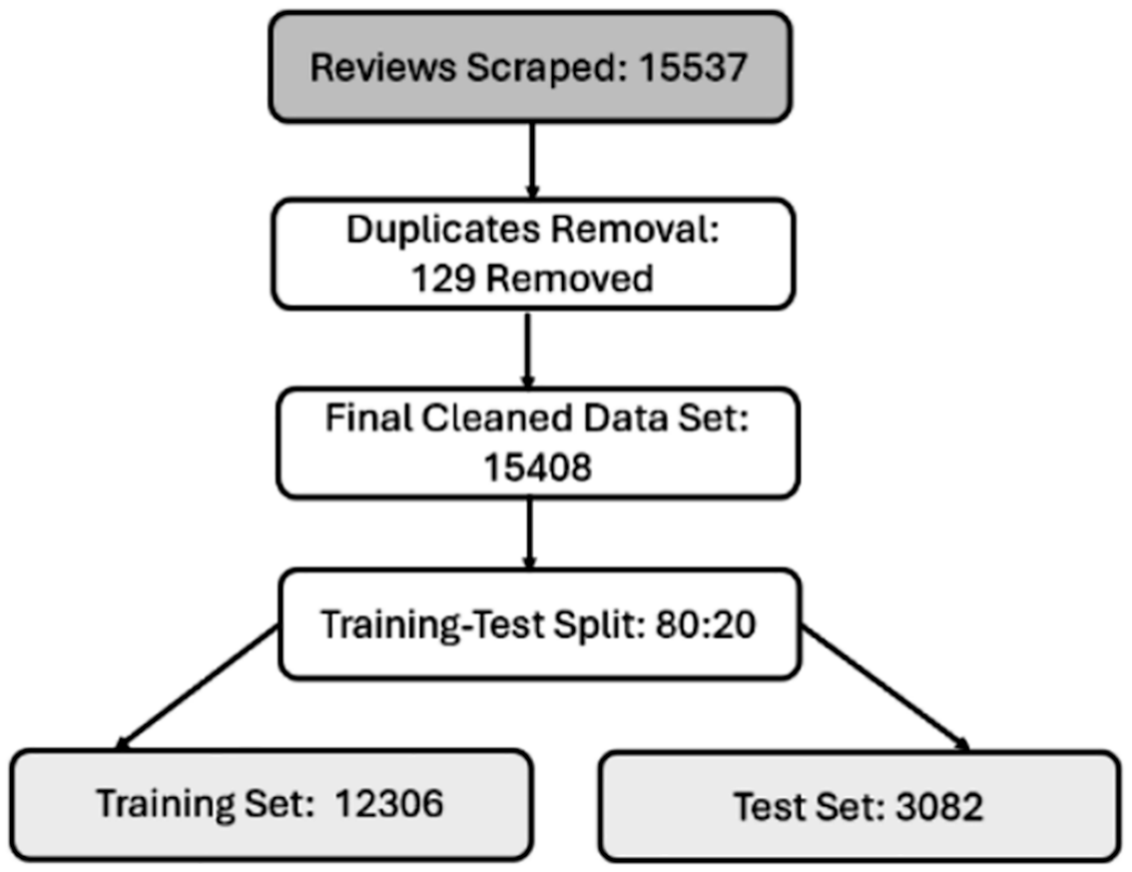
End-to-end review data flow.

Furthermore, topic modeling techniques, such as latent Dirichlet allocation (LDA) and word-frequency analysis, were applied to identify recurring themes. Multiple topic configurations (*K* = 5–20) were tested for the LDA model, with the final number of topics chosen based on the highest coherence score and consistent perplexity behavior. LDA preprocessing included lemmatization, removal of custom stop words, and bigram generation with a minimum document frequency threshold (min_df = 5). To improve subject separation, highly unusual and unduly frequent terms were filtered out. These stages ensured that the final subjects were stable, understandable, and data-driven rather than subjective. Feature extraction used traditional vectorization techniques, such as Bag-of-Words (BOW), TF-IDF, and hashing, as well as advanced word-embedding models, including Word2Vec, FastText, GloVe, and IndicBERT. After preprocessing, the cleaned dataset of 15,408 reviews was split into training and testing sets using an 80:20 ratio, yielding 12,326 and 3,082 samples, respectively (Figure 2).

To perform classification tasks, several machine learning models were employed, including logistic regression, SVM, random forest, XGBoost, and decision trees CatBoost, AdaBoost, LightGBM; deep learning models such as ResNet, BiLSTM and VGG16 were also investigated. VGG16 and ResNet were not applied to raw text or text converted to graphics. Instead, they were employed in a transfer-learning setup using dense word embeddings generated by Bag-of-Words, TF-IDF, Word2Vec, FastText, GloVe, and IndicBERT. These embeddings produce structured numerical matrices that convolutional layers can process, a method backed by previous NLP research on embedding grids (Li et al., 2020). With limited labeled data, using pretrained VGG16 and ResNet models enables faster feature extraction and improved generalization. This method avoids converting words to images; instead, each review is represented as a reshaped embedding tensor suited for convolution. Including these architectures enables comparisons between CNN-based transfer learning and NLP-specific models, as well as an evaluation of whether pretrained convolutional networks offer useful hierarchical features for text classification (Wang and Li, 2022). Ultimately, the model’s effectiveness was assessed using recall, accuracy, precision, and F1 score, ensuring the effective categorization of user review sentiments and theme analysis.

### 3.3 Data description

Our dataset included Google Play Store reviews of seven Indian RBI-approved lending platforms: 5Paisa, Faircent, i2ifunding, LenDenClub, CashKumar, Lendbox, and IndiaMoenyMart. The Google Play Store was the largest online market for mobile apps with over 2.6 million free and premium apps available as of May 2025 (Pamplona, 2022). Accordingly, we gathered this data using a site-scraping technique. The data file includes the user’s name, time and date, thumbs-up, written comments, and a rating on a scale of 1 to 5. Based on this, we divided the rating into three categories: positive (three or more), negative (below two), and neutral (more than two) (Pamplona, 2022; Wang, 2015) (Table 3).

**Table 3:** Platform-wise compilation of collected user reviews.

S. no.	Name of platform	Reviews
1	5Paisa	5,000
2	Faircent	3,295
3	i2ifunding	2,133
4	Lenden club	3,304
5	Cash Kumar	764
6	Lend box	761
7	India Moenymart	151
8	TOTAL	15,408

### 3.4 Data pre-processing

Preprocessing is an essential stage in machine learning and deep learning workflows, especially when handling unstructured user review data. This study scraped reviews from the Google Play Store using Python tools, capturing details such as username, timestamp, star rating (1–5), number of likes, and written comments. The raw text was cleaned by removing non-ASCII characters, HTML tags, URLs, emojis, punctuation, special symbols (@, #, %, etc.), and digits. All text was then converted to lowercase for consistency. Tokenization was applied to split sentences into individual words (e.g., “Loan process is fast” → [“loan,” “process,” “is,” and “fast”]). Common stop words like “the,” “is,” and “in” were removed using a tool, NLTK Lemmatization, to downsize words to their most basic form (e.g., “running” → “run”). In this study, sentiment labels were determined directly from user star ratings rather than through manual annotation or lexicon-based sentiment approaches. This approach has been widely used in previous studies (Thelwall et al., 2010; Pagolu and Majhi, 2016) because the numerical star rating is an explicit indication of user sentiment provided at the time of review submission. It provides a more objective reflection of the user’s overall contentment or dissatisfaction than textual polarity, which can be unclear or influenced by linguistic variances. Using rating-based sentiment tagging reduces subjectivity and ensures consistency across the dataset, preventing misclassifications caused by sarcasm, mixed viewpoints, or casual language in the review text. Reviews were categorized as positive (>3), neutral (=3), or negative (<3) based on ratings. A final manual review ensured the cleaned data preserved the intent and quality of the original content. To ensure data quality and consistency, the dataset was preprocessed multiple times. Missing or null reviews were removed, content was standardized to lowercase, and undesirable components such as URLs, punctuation, numerals, emojis, and special characters were removed. Stopwords were filtered using the NLTK package, and words were reduced to their base form using lemmatization. To prevent redundancy and bias in model training, duplicate reviews identified by identical text content were removed using the Python drop_duplicates () method.

### 3.5 Operationalization of theoretical constructs

This study includes TAM, UTAUT, SERVQUAL, and the Trust-Risk paradigm into the analytical design by transforming their basic conceptions into measurable text features. Perceived usefulness, ease of use, trust, responsiveness, reliability, and perceived risk were predefined as aspects expected in user evaluations and operationalized using topic-model probabilities, keyword clusters, and sentiment-based expressions. These constructs were extracted using two methods: topic modeling, which offered numerical indicators of construct salience, and supervised keyword dictionaries, which capture explicit occurrences of theory-related ideas. The generated variables were utilized as input features in machine learning models to investigate how each component correlates with sentiment and ratings. Random forest, XGBoost, and deep learning networks are examples of multivariate classifiers that incorporate topic weights and lexicon counts. Model interpretability methods, such as feature importance and SHAP analysis, quantified each construct’s impact, allowing theory-driven relationships to be validated within the ML framework rather than interpreted retrospectively.

## 4 Results

### 4.1 Analysis of words

**Figure 3:**
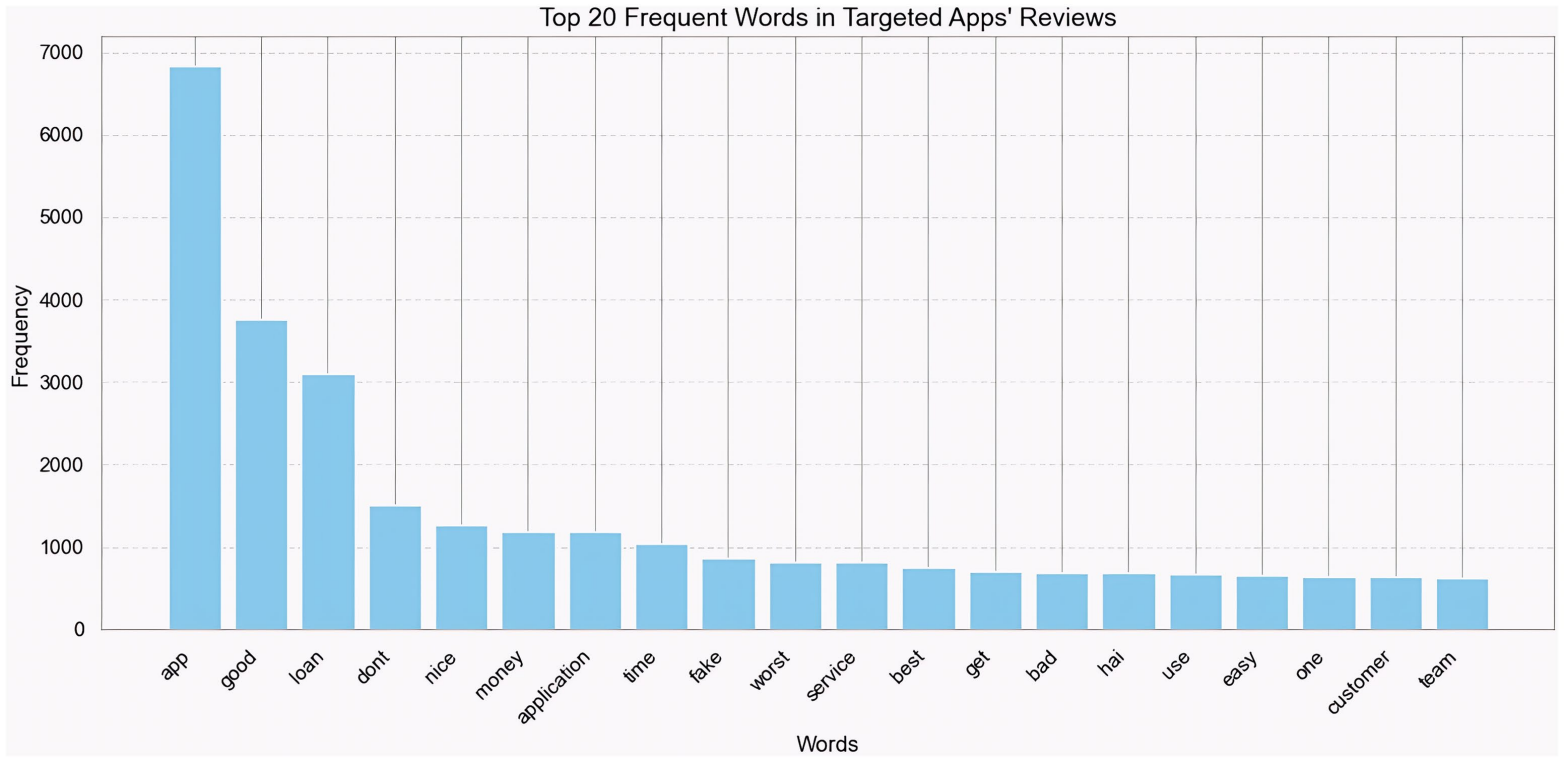
Most commonly used words in the review dataset.

Word analysis is a fundamental technique in text mining and natural language processing, enabling the extraction of insights from unstructured text. By analyzing word frequency and distribution, it is possible to find significant terms, recurring motifs, and underlying issues (Feldman et al., 2007). This method exposes patterns and linguistic trends that would otherwise be overlooked in manual study (Liu, 2020). Furthermore, when used alongside visualization tools such as word clouds or frequency histograms, word analysis can help evaluate public opinion, customer feedback, or social media content (Weiss et al., 2005) (Figure 3).

The top 20 terms used in user evaluations of the chosen apps are displayed in the bar chart. The top three terms on the list are “app” (6,800), “good” (3,800), and “loan” (3,200), indicating that users regularly leave comments on the application as a whole, offer compliments, and discuss loan-related aspects. Terms that convey a generally positive attitude and contentment with the app’s services include “nice” (1,400), “money” (1,300), “application” (1,200), and “easy” (730). Conversely, words such as “bad” (780), “fake” (1,000), “worst” (950), and “do not” (1,700) indicate user apprehension and unfavorable experiences. Additionally, references to “service” (900), “customer” (710), and “team” (700) reflect opinions about customer service and support interactions.

### 4.2 Topic modeling

Topic models are methods for identifying hidden themes in text, with Latent Dirichlet Allocation among the most widely used topic modeling approaches. Previous studies have demonstrated that topic models outperform more traditional clustering-based methods (Wei and Croft, 2006). Topic modeling is a useful approach for identifying textual groupings in huge collections (Liu, 2013). LDA, a widely used topic modeling algorithm, is effective in revealing latent semantic structures in text (Hofmann, 1999). Therefore, this study follows a research design that begins by preprocessing the raw textual data, then applies LDA to extract latent patterns, and finally integrates the LDA results with existing metadata for further analysis and visualization. Moreover, using the Gensim package in Python, we identified the following 11 topics (Table 4).

**Table 4:** Summary of extracted LDA topics with key terms and illustrative examples.

S.no.	Name of the topic	Frequency	Top keywords	Example review	Topic information
1	App interface	70	App, open, crash, slow, update, login, screen	The app keeps freezing every time I try to open it	Experience regarding the interface of an application.
2	Application experience	172	Experience, helpful, easy, useful, smooth, service	Good experience. The app works fine for my needs.	Overall satisfaction with the utility and usability of the application.
3	CIBIL and credit score	139	Cibil, score, report, affect, drop, enquiry	After installing the app, my CIBIL score suddenly dropped.	CIBIL score impact after installation and use of the application
4	Customer service	114	Support, help, call, response, team, contact	No one responds when I reach out to customer support	The customer service and verification team’s actions and responses
5	Document verification	286	Document, upload, verify, KYC, submit, approval	Verification was quick, and the documents were accepted easily	Ease of document verification
6	Loan process	38	loan, apply, amount, approve, process, interest	The loan process was simple, and the amount was credited quickly	Experience related to the easy loan money platform
7	Loan rejection	97	Reject, eligibility, application, declined	Applied twice and still got rejected without any reason	Loan application rejection and disapproval.
8	Login issues	81	login, error, password, access, problem	I cannot log in after the latest app update	Experience reentering the application after exiting it
9	OTP and verification	68	OTP, code, number, receive, verification	Not receiving OTP even after multiple attempts	Overall impression upon obtaining OTP
10	Repayment and EMI	252	Repay, EMI, return, investment, due, payment	Repayment was smooth, and EMI reminders were on time	Experience regarding returns on investment.
11	Withdrawal	135	withdraw, bank, transfer, amount, delay	Withdrawal took longer than expected to reflect in my account	Experience with money withdrawal

#### 4.2.1 Topic frequency overview

The significance of the subjects fluctuated over the dataset. Document verification, payback concerns, and the overall application experience were the most frequently mentioned topics. Loan processing, loan rejections, login difficulties, and interface issues occurred regularly.

#### 4.2.2 Topic quality and diagnostics

After analyzing various topic sizes, the final LDA model developed a coherent structure. The chosen model achieved a high coherence score, indicating well-defined and interpretable motifs. Review excerpts with the greatest topic likelihood were personally reviewed to ensure topic consistency, and noisy or overlapping clusters were reduced using preprocessing techniques such as bigram generation and frequency-based token filtering.

#### 4.2.3 Platform-specific salience

Topic distribution varies across platforms. Loan denials, login issues, and customer service concerns were more common at Lendbox, CashKumar, and Faircent. Document verification, repayment, and withdrawal-related discussions were more prevalent on IndiaMoneyMart and i2iFunding. Topics such as interface and application experience were discussed across all platforms, though 5Paisa and LendenClub received the most attention. Platform-specific processes and operational practices impact user concerns and experiences, as demonstrated by these distinctions.

### 4.3 Evaluation of sentiment

A computational technique called sentiment analysis is used to identify and categorize textual opinions about a person, an event, or a product as neutral (=3), negative (<3), or positive (>3) (Alam and Yao, 2019). In this study, we applied sentiment analysis methods to classify users’ online text comments as either good or negative accounts of their experiences with lending apps. Sentiment labels were assigned based on star ratings, as they provide a clear and consistent representation of customer satisfaction. Text-rating comparisons revealed strong alignment, with positive terms appearing in 4-5-star reviews and complaints dominating in 1-2-star reviews. A human-labeled subset supported this pattern, and inter-rater reliability (Cohen’s kappa) confirmed consistent judgment among annotators (Al-Natour and Turetken, 2020). Rating-based labeling also matched the polarity created by VADER and transformer models, particularly for obvious positive and negative examples. Sensitivity tests utilizing stricter criteria (e.g., ≥4 for positive) yielded consistent results, indicating the reliability of rating-based sentiment classifications (Noori, 2021). To evaluate user feedback, we use sentiment analysis to assess reviews; it is superior to traditional techniques (Greaves et al., 2013). Platform comparisons are descriptive and based purely on the dataset’s observed distribution of feelings and reviews. Because the study is based on user-generated reviews, the differences across apps should not be considered statistically significant. These results reflect patterns observed in accessible reviews, and variances may be driven by factors such as review volume, app age, update history, or review-soliciting techniques.

#### 4.3.1 Overall sentiment analysis of the combined data

**Figure 4:**
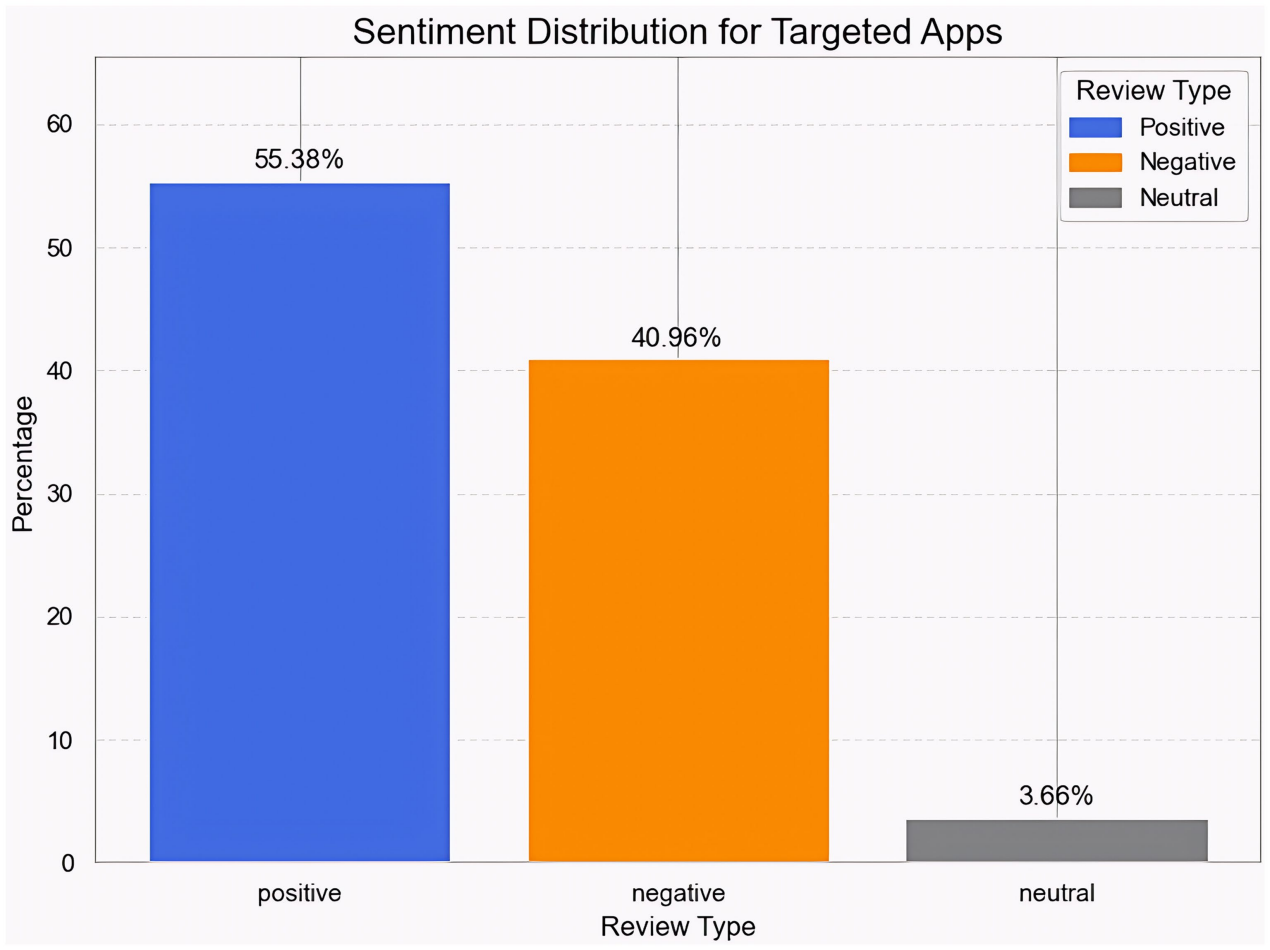
Sentiment distribution of the merged review dataset.

The sentiment classification of user reviews and comments was carried out in this study utilizing a rule-based methodology based on the user-provided numerical ratings. Reviews that scored higher than three were classified as positive, reviews that scored lower than three as negative, and reviews that scored three or above were classified as neutral (Sherman, 2014; Moraes et al., 2013). Without requiring pre-trained models or external sentiment analysis tools, this approach enabled the creation of a systematic, interpretable framework for sentiment categorization (Figure 4).

The sentiment distribution for targeted apps shows a predominantly positive user experience, with 55.38% of reviews rated positive. However, a sizable proportion, 40.96%, reflects negative sentiment, indicating widespread dissatisfaction among users. Neutral feedback accounts for only 3.66%, indicating that consumers prefer to share strong opinions rather than moderate or uninterested reviews. This division in attitude underscores the need to address users’ concerns while preserving and enhancing features that have already been well received. Overall, the finding demonstrates the importance of sentiment analysis in capturing user perceptions and guiding improvements in app quality and satisfaction.

#### 4.3.2 Overall sentiment analysis of applications

**Figure 5:**
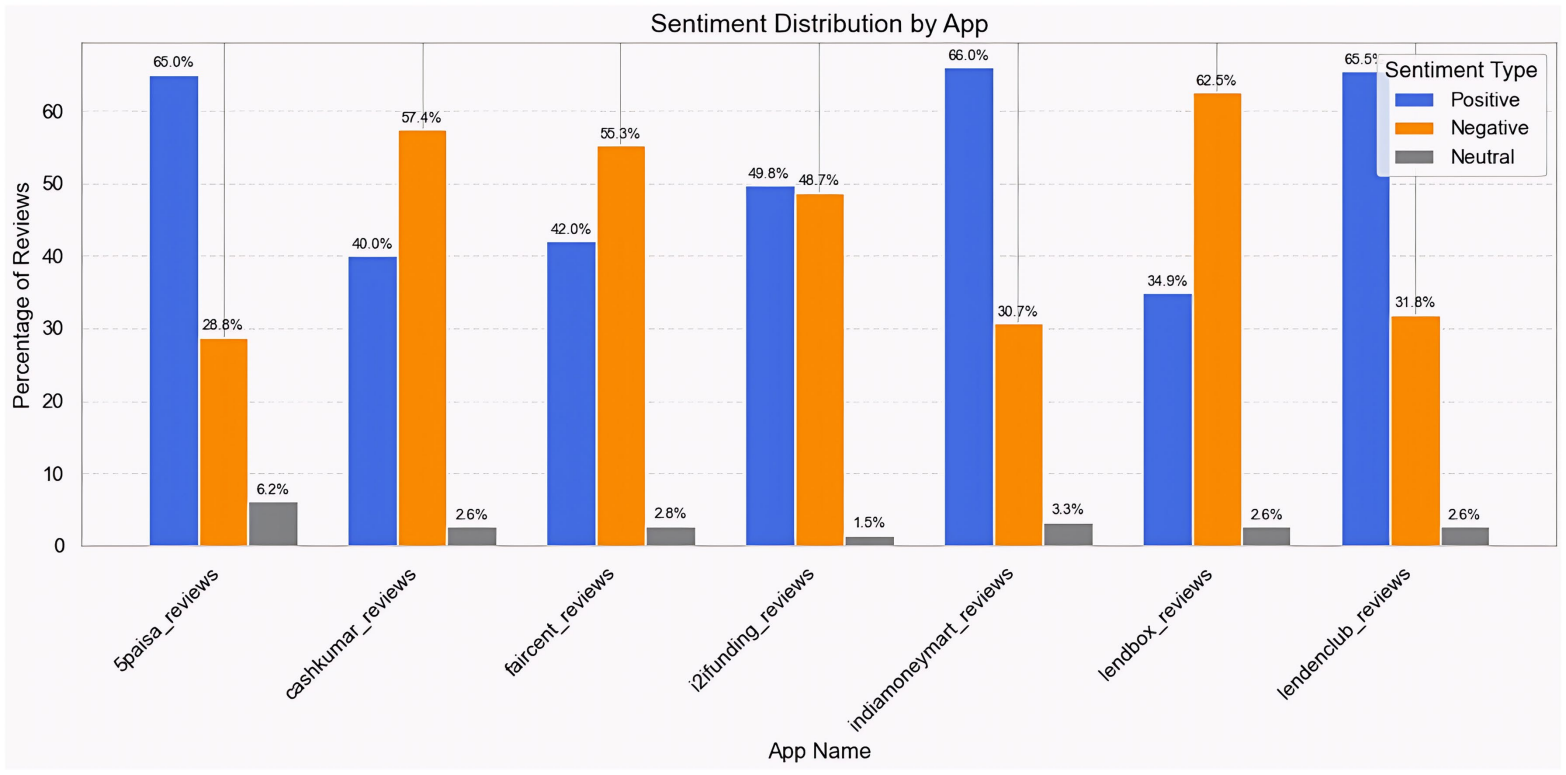
Sentiment comparison across the seven lending apps.

Better comprehension across all lending platforms, India Money Mart receives the most positive feedback, with 66.0% of reviews indicating positive sentiment. It is closely followed by LendClub (65.5%) and 5paisa (65.0%), indicating high user performance and a user-friendly, smooth lending process. On the other hand, apps such as Cash Kumar (40.0%) and Lendbox (34.9%) had the fewest positive reviews, indicating poor user satisfaction. In terms of negative sentiment, Lendbox had the highest proportion at 62.5%, followed by CashKumar at 57.4% and Faircent at 55.3%, which might be attributed to difficulties or unfavorable lending terms. Meanwhile, India Money Mart and 5paisa had comparatively few negative reviews at 30.7 and 28.8%, respectively, confirming their positive public image. Neutral sentiment was low across all platforms, with 5paisa at 6.2% and i2ifunding at 1.5%, indicating that consumers often expressed strong opinions. Overall, India Money Mart and 5paisa have the finest sentiment balance, whereas Lendbox has the most unfavorable customer experience and might benefit from strategic customization (Figure 5).

#### 4.3.3 Topic-based sentiment analysis

**Figure 6:**
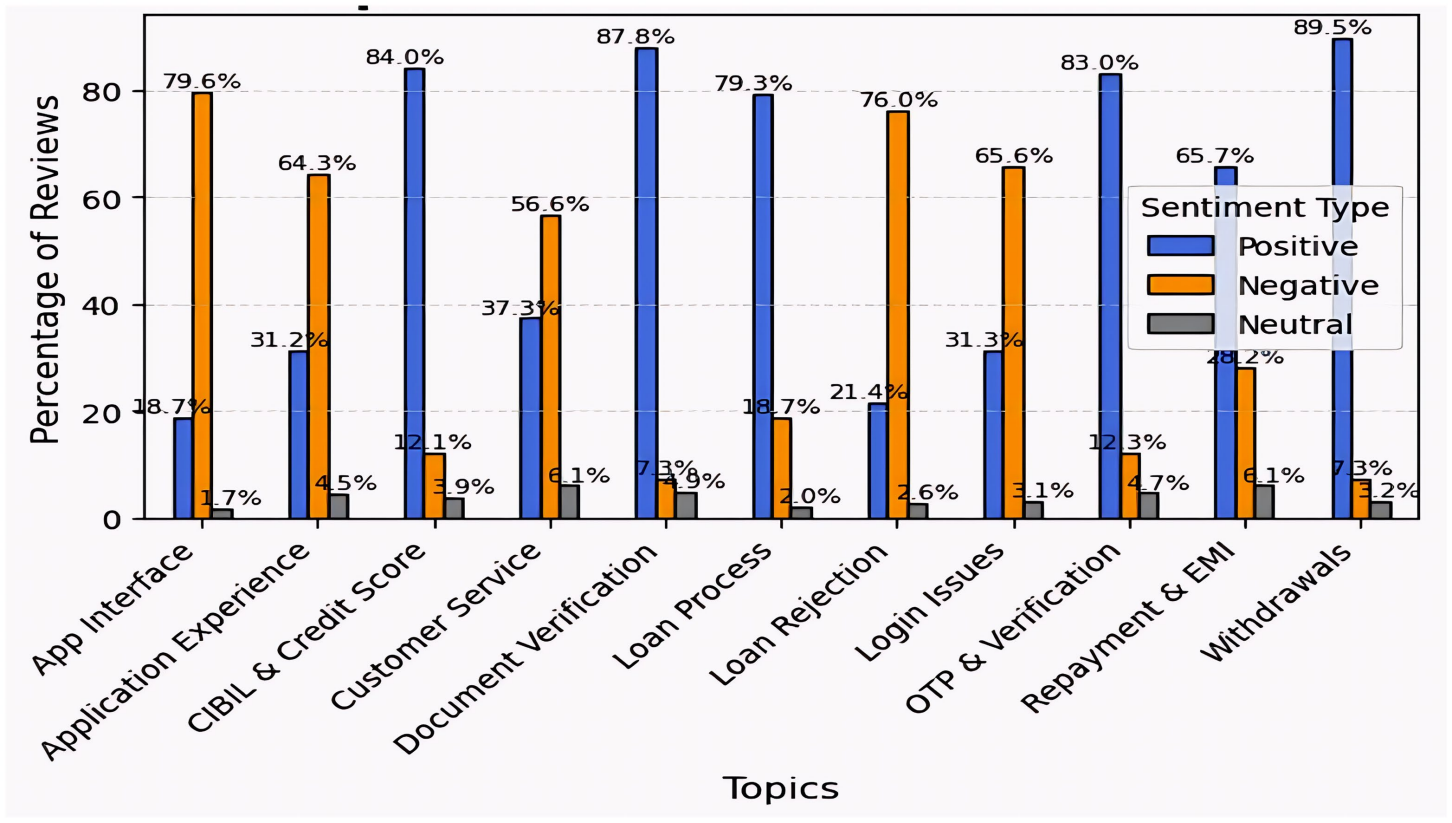
Topic-wise sentiment analysis of the merged dataset.

To learn more about user experience, we conducted sentiment analysis on topics retrieved via topic modeling. This strategy helps developers and prospective users make better decisions. The results show significant variation in sentiment across services. Document verification (87.8%), withdrawals (89.5%), CIBIL score (84%), and OTP and verification (84.0%) received very excellent reviews from users, indicating that post-approval procedures are generally efficient and seamless. On the other hand, significant negative sentiments were expressed regarding the app interface (79.6%) and login rejection (76.0%). All elements received small neutral feedback. To improve overall user satisfaction and app retention, developers should immediately address issues with user onboarding and accessibility (Figure 6).

#### 4.3.4 Topic sentiment for individual apps

To better understand, each P2P lending app undergoes sentiment analysis on a specific topic. This study enables the classification of apps based on several criteria inferred from user sentiment. Furthermore, it helps identify areas for improvement by highlighting user-identified negative aspects.

##### 4.3.4.1 Topic sentiment analysis for 5Paisa

**Figure 7:**
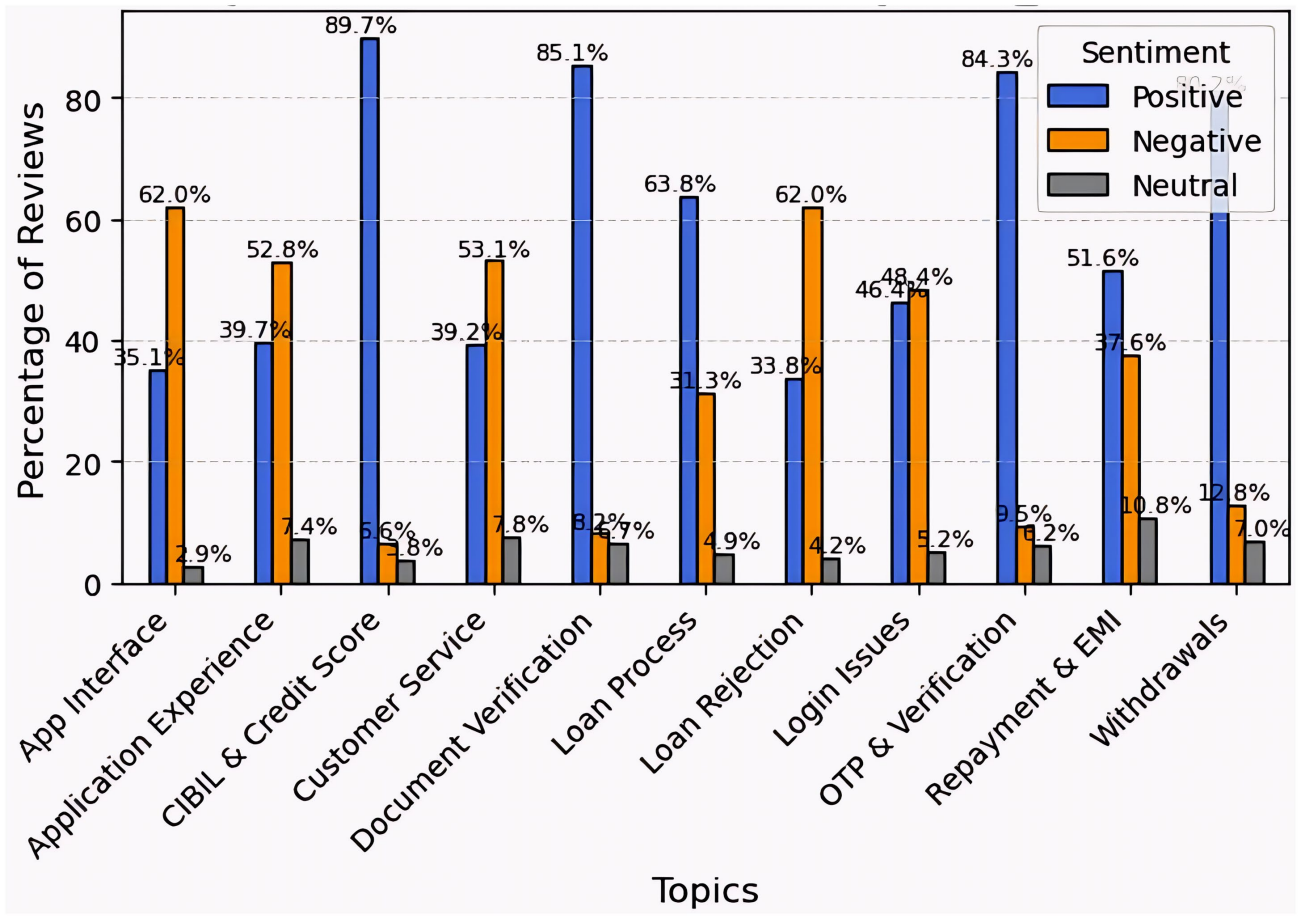
Topic-wise sentiment analysis for 5paisa.

The sentiment analysis of 5Paisa reviews reveals both advantages and disadvantages. Users were very satisfied with CIBIL and credit scores (89.7%), OTP and verification (84.3%), document verification (85.1%), and withdrawals (80.2%), suggesting consistent execution across core activities. However, the unfavorable opinion was high for the application interface (loan rejection: 62.0%; login troubles: 48.4%). The amount of neutral input was minimal. Overall, while the app excels in financial services, improving usability and login functionality would enhance the user experience (Figure 7).

##### 4.3.4.2 Topic sentiment analysis for Faircent

**Figure 8:**
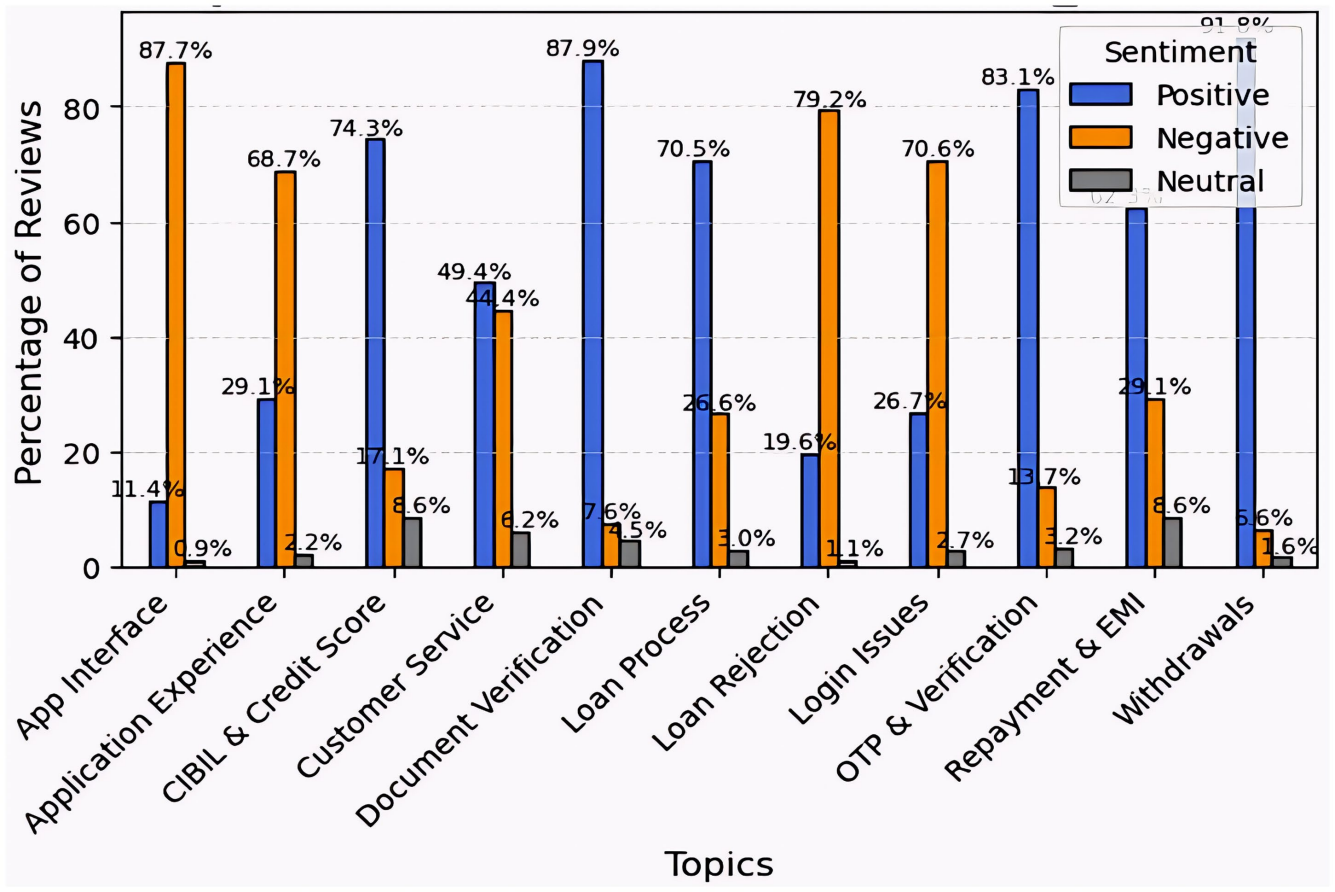
Topic-wise sentiment analysis for Faircent.

Faircent outperforms in essential lending activities, such as document verification (87.9%), withdrawals (91.8%), and OTP verification (83.1%), demonstrating high user satisfaction with its core financial services. However, the platform shows significant user-experience difficulties, including the app interface (87.7% negative sentiment), the application process (79.2%), the application experience (68.7%), and login issues (70.6%). While customer service received mixed feedback, most other areas elicited strong emotional responses, indicating substantial usability and technical problems. Overall, Faircent is dependable for transactions, but it needs to improve its user interface, onboarding process, and accessibility to increase overall user satisfaction (Figure 8).

##### 4.3.4.3 Topic sentiment analysis for i2iFunding

**Figure 9:**
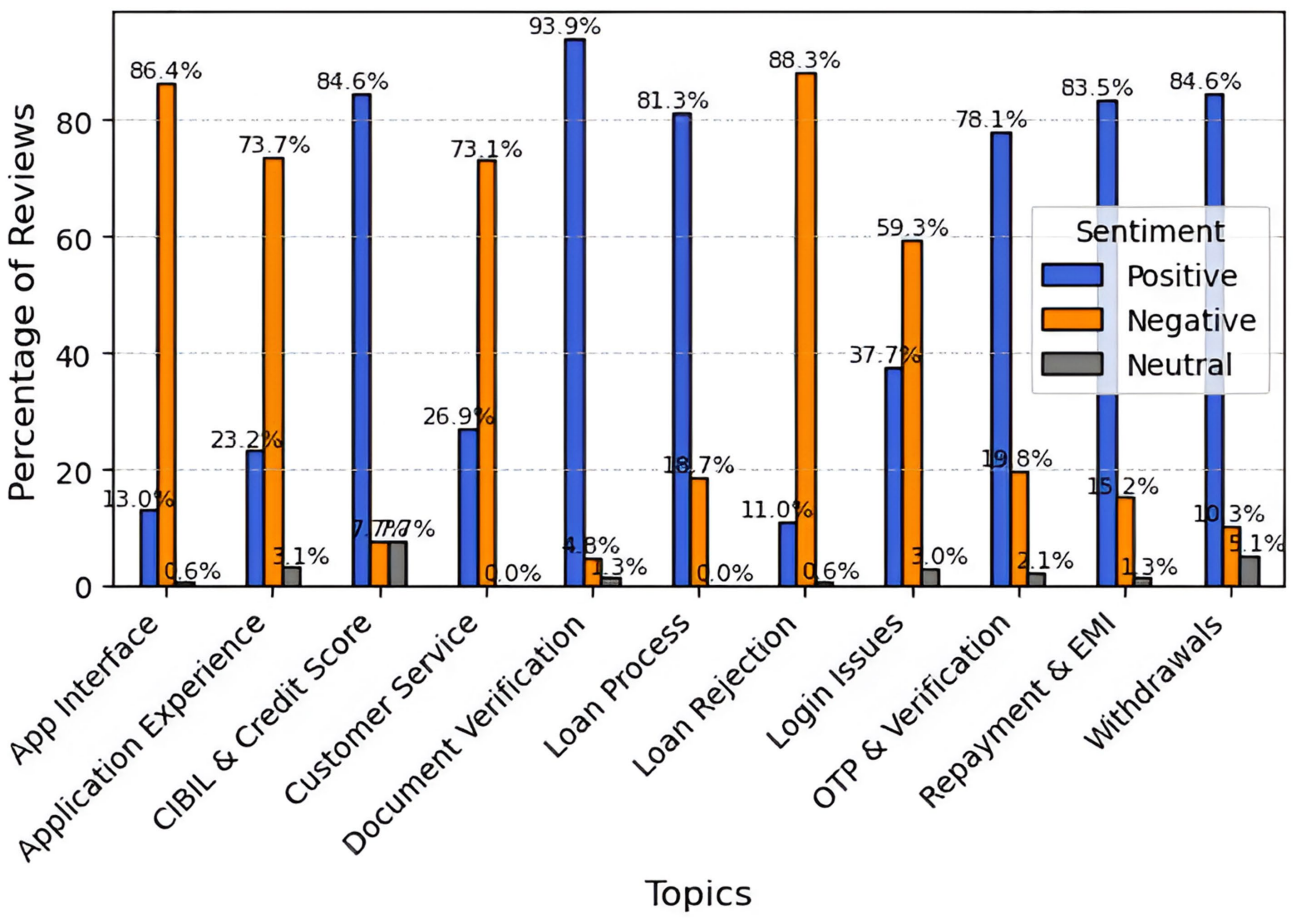
Topic-wise sentiment analysis For i2iFunding.

i2iFunding receives strong, favorable ratings for its basic services, including document verification (93.9%), withdrawals (84.6%), CIBIL and credit score checks (84.6%), and repayment and EMI processes (83.5%), indicating high user satisfaction with its financial operations. However, the platform is criticized for its app interface (86.4% negative sentiment), loan rejection experiences (88.3%), application experience and customer service (73%), and login issues (59.3%). These negative ratings point to notable usability and transparency concerns. Overall, the feedback highlights i2ifunding’s expertise in financial operations while also indicating the need for significant changes in interface design, accessibility, and user onboarding experience (Figure 9).

##### 4.3.4.4 Sentiment analysis for LenDenClub

**Figure 10:**
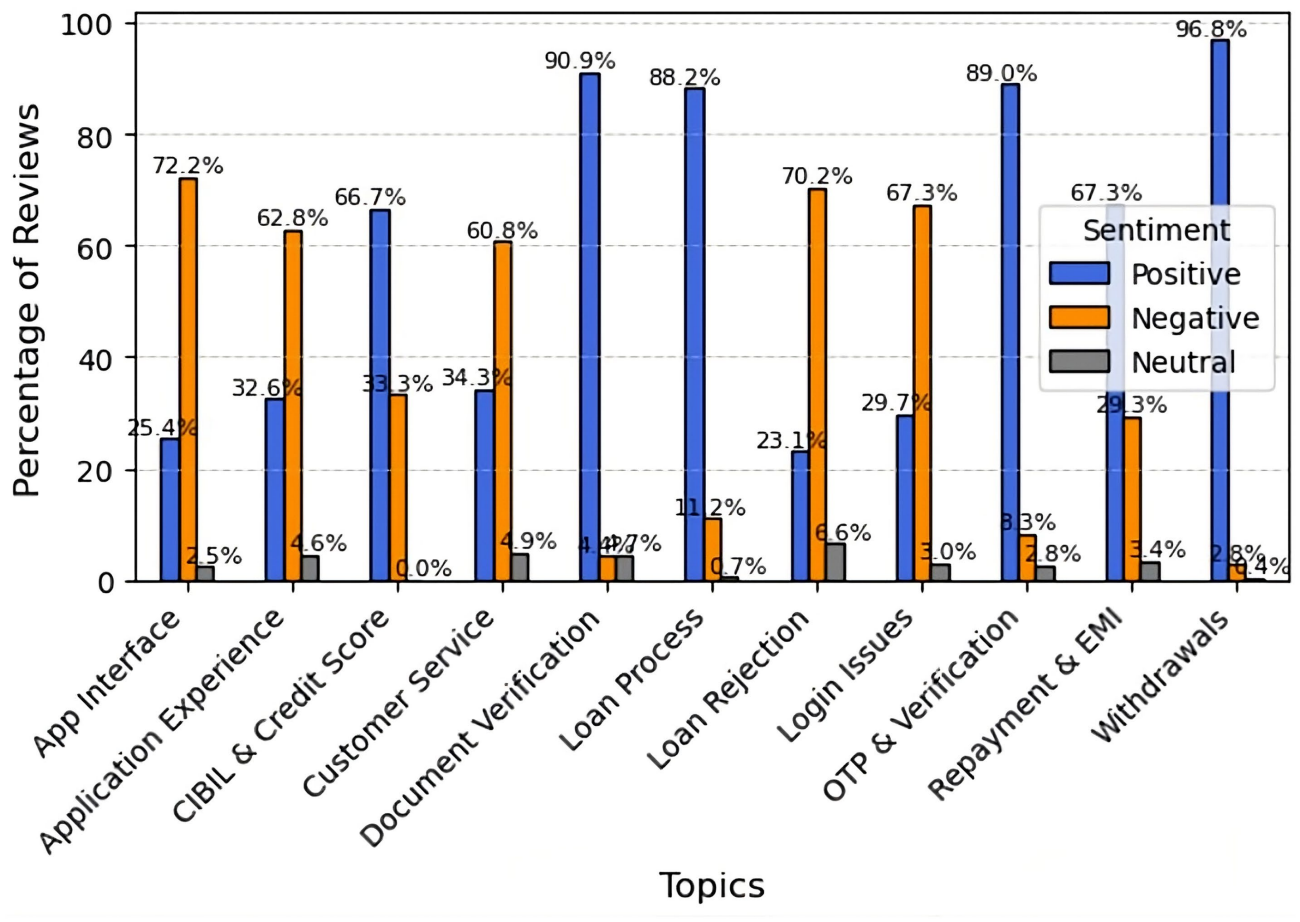
Topic-wise sentiment analysis for LenDenClub.

According to a sentiment analysis of LenDenClub evaluations, consumers reported highly positive experiences with withdrawals (96.8%), document verification (90.9%), OTP verification (89.0%), and the loan process (88.2%). However, unfavorable opinions predominated across the app interface (72.2% negative), loan rejection (70.2%), login issues (67.3%), and overall app experience (62.8%), indicating user dissatisfaction. Although customers value the basic lending features, technological issues and early-stage procedures appear to require improvement, revealing both strengths and weaknesses in the overall user experience (Figure 10).

##### 4.3.4.5 Topic sentiment analysis for CashKumar

**Figure 11:**
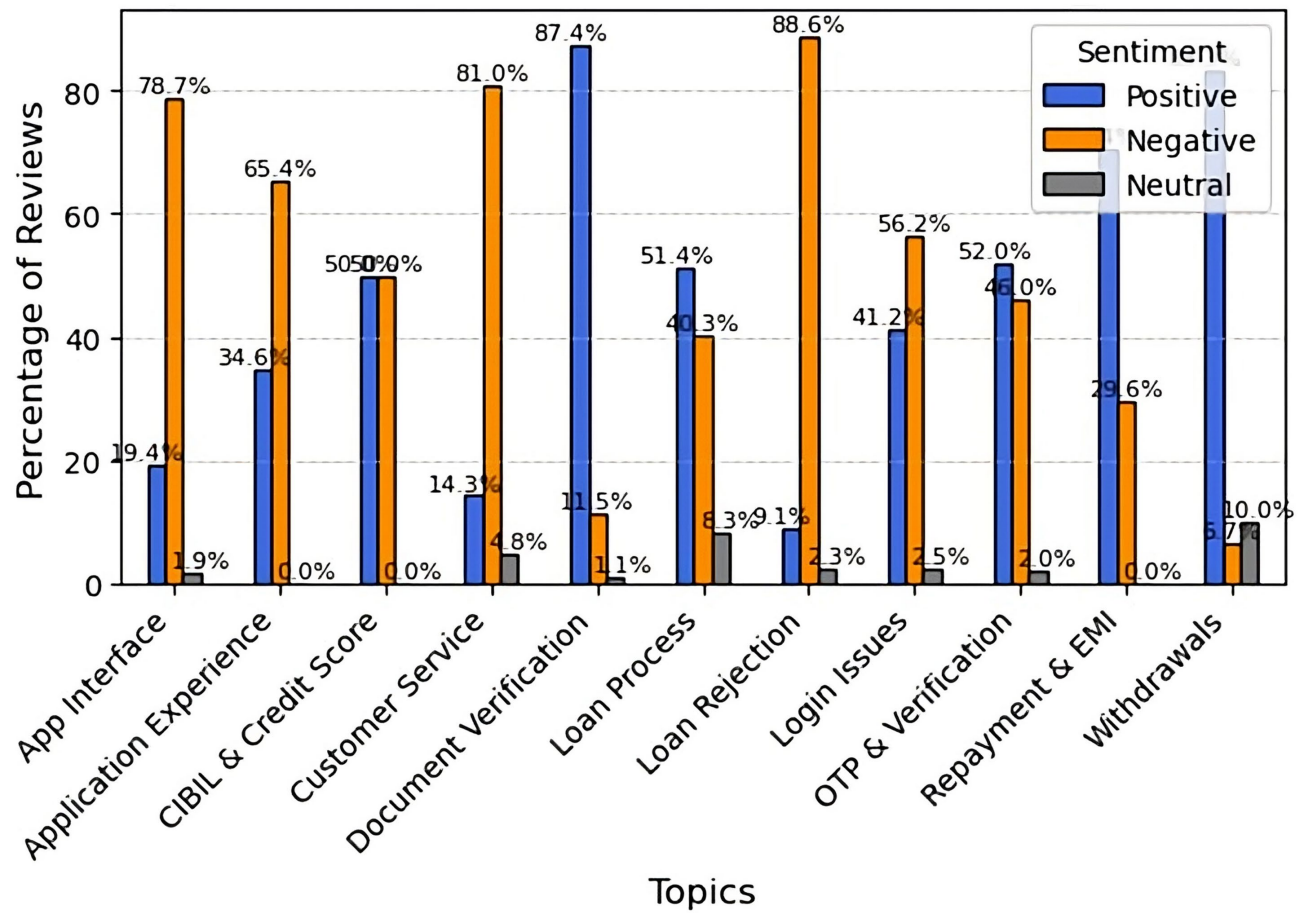
Topic-wise sentiment analysis for CaskKumar.

The sentiment analysis of CashKumar reviews reveals highly unfavorable comments, particularly regarding loan rejection (88.6%), customer service (81.0%), the interface (78.7%), and the application experience (65.4%), indicating issues with usability, support, and loan approvals. Login difficulties (56.2%) were also criticized. On the positive side, users rated document verification (87.4%), withdrawals (83.3%), repayment (70.4%), and the loan process (51.4%) favorably, with mixed feedback on CIBIL and credit score checks. Neutral feedback was minimal, indicating a strong overall consensus among users. Although CashKumar performs well in loan disbursements, repayments, and withdrawals, it needs to improve its user interface, customer service, and transparency in loan approvals to enhance user satisfaction (Figure 11).

##### 4.3.4.6 Sentiment analysis for Lendbox

**Figure 12:**
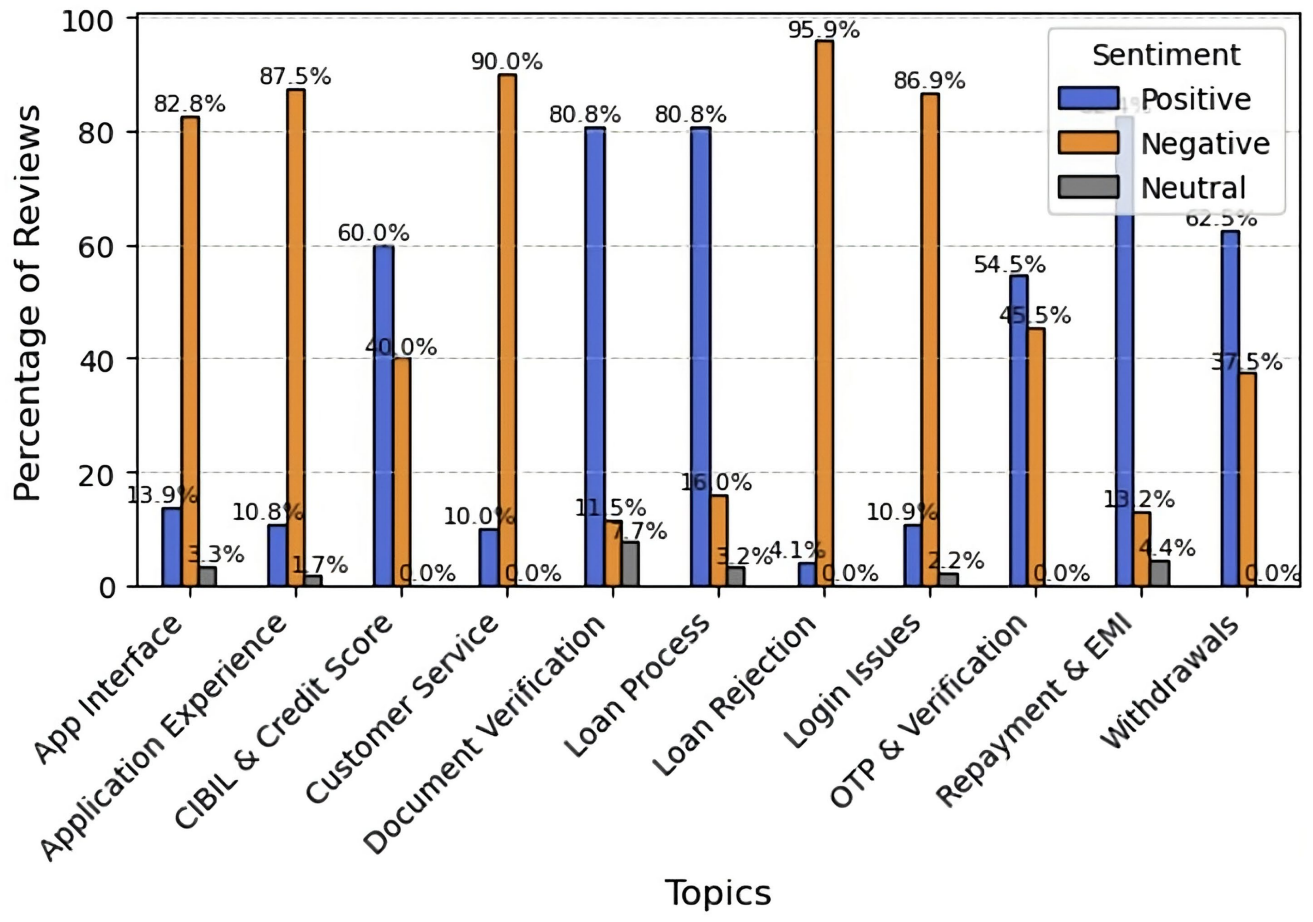
Topic-wise sentiment analysis for Lendbox.

Lendbox performs well across financial operations, including loan repayment and EMI (82.4%), loan processing (80.8%), document verification (80.8%), and credit score handling (60%). Nevertheless, the platform receives strong criticism for loan refusal (95.9% negative), customer service (90% negative), app experience (87.5% negative), login issues (86.9% negative), and the app user interface (82.8% negative). Opinions regarding OTP verification are mixed, with 54.5% positive and 45.5% negative sentiment. Although Lendbox performs reliably in core learning operations, its overall dependability and user satisfaction are limited by technological issues and an inadequate user interface (Figure 12).

##### 4.3.4.7 Sentiment analysis for IndiaMoneyMart

**Figure 13:**
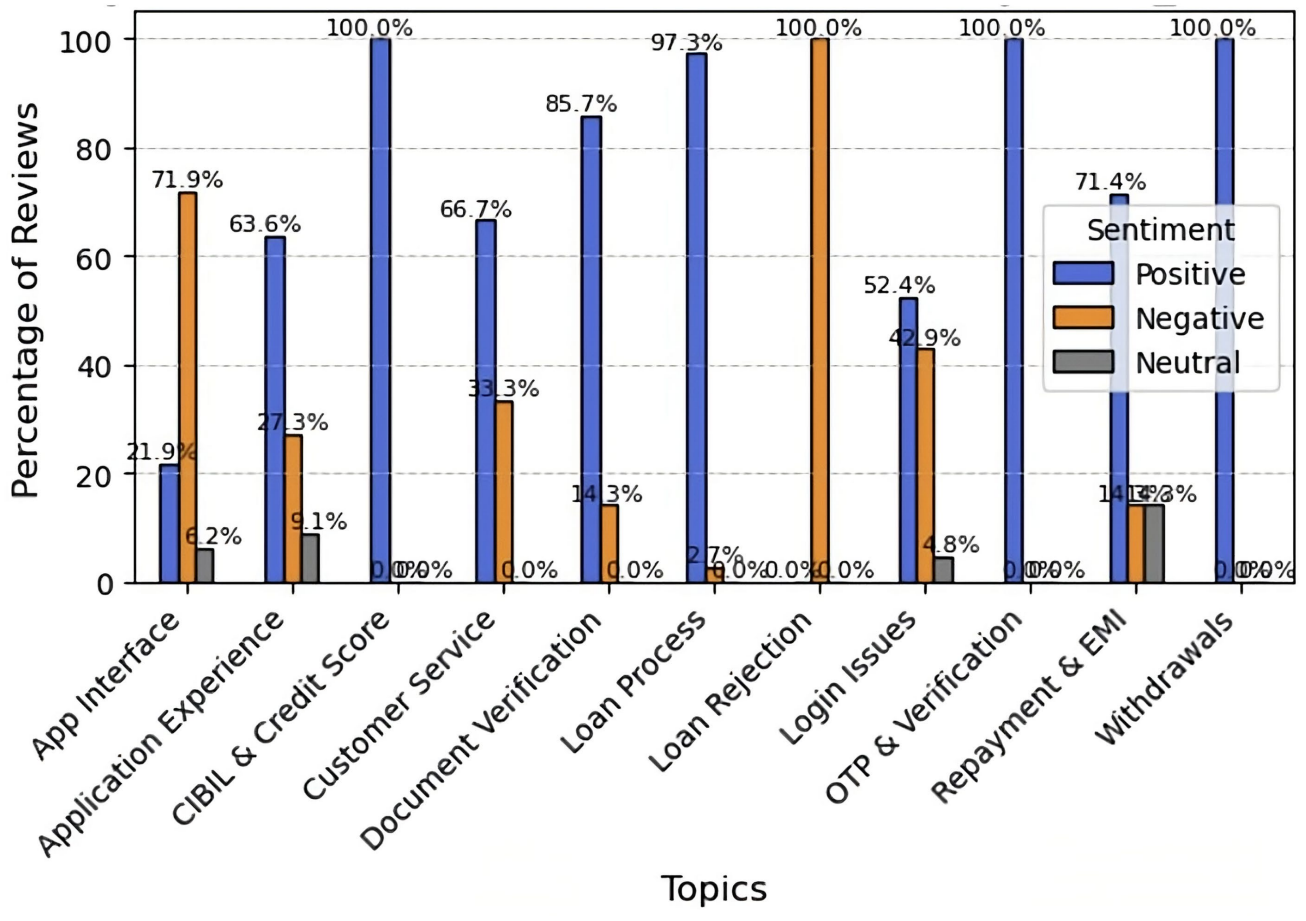
Topic-wise sentiment analysis for IndiaMoneyMart.

IndiaMoneyMart receives exceptionally positive feedback on its financial operations, including CIBIL score checks, OTP and verification, and withdrawals (all 100%), demonstrating strong user confidence in its core services. The loan process (97.3%), document verification (85.7%), repayment (71.9%), customer service, and overall app experience also receive positive feedback. However, users are dissatisfied with loan rejections (100% negative) and the app interface (71.9% negative), indicating clear areas for improvement. Overall, IndiaMoneyMart stands out as a dependable, high-performing loan platform, though its user interface and login functionality need improvement (Figure 13).

### 4.4 The extraction of features

Feature extraction is essential to the suggested methodology for evaluating user reviews of digital lending applications regulated by the RBI, as it transforms unstructured textual data into representations useful for ML and DL algorithms. In this study, we use traditional vectorization techniques and advanced word embedding approaches to capture both semantic and structural information.

Traditional methods, such as Bag-of-Words, TF-IDF, and hashing algorithms, are initially employed. BOW measures the frequency of words such as fault-related phrases, treating each text as a collection of words regardless of structure or word order (Zhao et al., 2022). TF-IDF improves on BOW by calculating the relevance of a word in a document relative to the entire corpus, resulting in a more informative vector representation (Ahuja et al., 2019). Hashing uses hash functions to convert words into fixed-length sparse vectors, which is computationally efficient. However, while these algorithms capture basic text structure, they sometimes fail to preserve contextual meaning, have excessive dimensionality, and have limited accuracy (Singh and Gupta, 2022). To overcome these drawbacks. Advanced word-embedding methods, such as word2Vec, GloVe, FastText, and IndicBERT, are used. Word2Vec learns high-quality word representations from context, enabling the model to interpret word relationships (Alshari et al., 2020). FastText goes beyond this by including subword information, which is especially valuable in morphologically rich languages (Yao et al., 2020). GloVe constructs word vectors using global co-occurrence statistics and performs classification tasks using backpropagation neural networks (Mahmood and Abdulazeez, 2019). IndicBERT, a transformer-based multinational model trained on various Indian languages, improves the system’s ability to understand regional language nuances. In morphologically rich languages, models such as GloVe construct word vectors using global co-occurrence statistics, providing a semantic foundation for performance classification (Sankalp et al., 2024). By integrating both conventional and current feature extraction techniques, the framework facilitates a more comprehensive and contextualized examination of user feelings, often compared with traditional methods and subjects in reviews of digital lending apps, ultimately enhancing model performance and insight generation.

#### 4.4.1 ML algorithms

We are applying machine learning to customer reviews for improve analysis by enabling automation, increasing accuracy, and delivering valuable insights. Unlike rule-based techniques, ML models can correctly identify new, unread reviews as positive or negative. This enables the organization to monitor real-time sentiment, discover issues such as bugs or UI complaints, and assess app performance following changes. ML also powers advanced applications that recognize users’ tone and systems that identify unexpected increases in negative comments. Furthermore, it supports customer segmentation and turnover prediction, enabling app developers to optimize the user experience and make informed decisions based on user feedback patterns (Table 5).

**Table 5:** Manual vs. machine learning analysis.

Method	Limitations without ML	Benefits of ML
Sentiment analysis	Rule-based (VADER or rating-based) systems are unable to adjust to humor or new expressions.	ML uses complex conversations to learn.
Topic modeling	Demonstrates what people discuss, not how frequently or how intensely.	ML can categorize, forecast, or order reviews based on emotion or topic.
Visuals	Excellent for summarizing, but lagging	ML enables dynamic forecasting of future reviews.

#### 4.4.2 DL algorithms

Without the need for handcrafted features, deep learning models automatically discover complex patterns and contextual correlations, making them ideal for analyzing text data such as user reviews. DL techniques like LSTM and BERT, unlike classical models, capture word order, semantics, and deeper meaning, thereby improving sentiment classification accuracy. In addition to supporting real-time analysis and scaling to large datasets, these models are ideal for gaining insight into user sentiment and improving decision-making in app review analytics.

### 4.5 Evaluation of prediction model effectiveness

To evaluate the classification models’ performance, we use F1 score, recall, accuracy, and precision as evaluation metrics. Accuracy indicates total correctness, whereas precision represents the proportion of actual positive predictions. Recall represents the model’s ability to recognize true positives, and the F1 score provides a comprehensive view of the model’s effectiveness by balancing accuracy and recall, particularly with imbalanced data. This evolution is conducted across various ML models using BOW, TF-IDF, hashing, and DL models such as Word2Vec, FastText, GloVe, and Indic-BERT embeddings (Table 6).

**Table 6:** Performance of ML and DL models using the BOW technique.

Predictive algorithm	Classification accuracy	Precision	Recall	F1 measure
Machine learning	–	–	–	–
1 Logistic regression	0.85	0.87	0.85	0.86
2 Random forest	0.85	0.85	0.88	0.86
3 Extreme gradient boosting	0.86	0.86	0.86	0.86
4 Decision tree	0.83	0.84	0.81	0.82
5 Support vector machine	0.86	0.85	0.88	0.86
6 CatBoost	0.87	0.84	0.87	0.85
7 AdaBoost	0.84	0.82	0.84	0.82
8 LightGBM	0.86	0.83	086	0.85
Deep learning	–	–	–	–
9 VGG16	0.87	0.84	0.87	0.85
10 ResNet	0.63	0.61	0.63	0.60
11 BiLSTM	0.87	0.84	0.87	0.85

The table compares the performance of several machine learning and deep learning models with Bow across key parameters, including accuracy, precision, recall, and F1. CatBoost and XGBoost are the most accurate machine learning classifiers (0.87 and 0.86, respectively), followed by SVM, random forest, LightGBM, and logistic regression, all of which perform well across various criteria. AdaBoost also performs well, although at a significantly lower level. In deep learning models, VGG16 matches CatBoost’s accuracy (0.87), while BiLSTM performs similarly (0.87). Overall, both advanced ML models, such as CatBoost, and the deep learning model VGG16, achieve top results, though ResNet’s low scores underscore the need for careful model selection (Table 7).

**Table 7:** Performance of ML and DL Models using the TF-IDF technique.

Predictive Algorithm	Classification accuracy	Precision	Recall	F1 measure
Machine learning	–	–	–	–
1 Logistic regression	0.86	0.86	0.88	0.86
2 Random forest	0.86	0.85	0.87	0.87
3 Extreme gradient boosting	0.86	0.84	0.86	0.85
4 Decision tree	0.83	0.81	0.83	0.82
5 Support vector machine	0.86	0.84	0.86	0.86
6 CatBoost	0.87	0.84	0.87	0.85
7 AdaBoost	0.84	0.82	0.84	0.82
8 LightGBM	0.87	0.84	0.87	0.85
Deep learning	–	–	–	–
9 VGG16	0.87	0.87	0.87	0.85
10 ResNet	0.61	0.61	0.61	0.57
11 BiLSTM	0.87	0.84	0.87	0.85

TI-FIDF shows that the two machine learning classifiers with the highest accuracies (0.87 each) are CatBoost and LightGBM. They are closely followed by SVM, random forest, XGBoost, and logistic regression, all of which have strong, comparable performances (0.86). The scores of AdaBoost and the decision tree are marginally lower. VGG16 stands out in deep learning for its high accuracy and balanced metric values (all 0.87 except the F1 score, which is 0.85). BiLSTM also exhibits robust results (0.87), comparable to the best models. While ResNet performs poorly, the majority of deep learning models perform well. This underscores the importance of choosing models that are appropriate for the data (Table 8).

**Table 8:** Performance of ML and DL models using the hashing technique.

Predictive algorithm	Classification accuracy	Precision	Recall	F1 measure
Machine learning	–	–	–	–
1 Logistic regression	0.81	0.84	0.81	0.82
2 Random forest	0.84	0.83	0.84	0.83
3 Extreme gradient boosting	0.85	0.82	0.85	0.84
4 Decision tree	0.77	0.75	0.77	0.76
5 Support vector machine	0.86	0.83	0.86	0.85
6 CatBoost	0.85	0.82	0.85	0.84
7 AdaBoost	0.80	0.77	0.80	0.78
8 LightGBM	0.85	0.82	0.85	0.83
Deep learning	–	–	–	–
9 VGG16	0.84	0.81	0.84	0.83
10 ResNet	0.61	0.58	0.61	0.59
11 BiLSTM	0.84	0.81	0.84	0.82

The table shows the performance of machine learning models that employ hashing algorithms. SVM achieved the best performance, with an accuracy of 0.86 and an F1 score of 0.85. XGBoost, CatBoost, and LightGBM were close behind, each obtaining an accuracy of 0.85 and an F1 score of 0.84. Random forest also performed well (0.84 accuracy, 0.83 F1 score). Logistic regression and AdaBoost achieved lower accuracies of 0.81 and 0.80, respectively. The decision tree achieved the lowest results (0.77 accuracy, 0.76 F1 score).

Among the deep learning models, VGG16 and BiLSTM performed best, achieving classification accuracies of 0.84 and F1 scores of 0.83 and 0.82, respectively. ResNet, however, performed poorly, with an accuracy of 0.61 and an F1 score of 0.59. Overall, the SVM, VGG16, and BiLSTM models outperformed the others (Table 9).

**Table 9:** Performance of ML and DL models using the Word2Vec technique.

Predictive algorithm	Classification accuracy	Precision	Recall	F1 measure
Machine learning	–	–	–	–
1 Logistic regression	0.87	0.84	0.86	0.85
2 Random forest	0.87	0.85	0.87	0.85
3 Extreme gradient boosting	0.87	0.83	0.87	0.86
4 Decision Tree	0.81	0.80	0.81	0.81
5 Support vector machine	0.87	0.83	0.86	0.84
6 CatBoost	0.87	0.84	0.87	0.85
7 AdaBoost	0.85	0.82	0.85	0.83
8 LightGBM	0.86	0.82	0.86	0.84
Deep learning	–	–	–	–
9 VGG16	0.86	0.83	0.86	0.84
10 ResNet	0.85	0.82	0.85	0.83
11 BiLSTM	0.86	0.82	0.86	0.84

According to the Word2Vec evaluation results, the majority of machine learning models, including logistic regression, Random forest, SVM, and CatBoost, achieved high classification accuracy (87%) and balanced precision, recall, and F1 scores. LightBGM closely followed with 0.86% accuracy, while XGBoost achieved the highest F1 score (0.86) for precision and recall. CatBoost performed exceptionally well in recall (0.87). AdaBoost and the decision tree showed comparatively lower performance. VGG16 and BiLSTM matched the top ML models, achieving 86% accuracy and an F1 score of 0.84, while ResNet trailed with 85% accuracy and an F1 score of 0.80. Overall, the most reliable algorithms were logistic regression, random forest, XGBoost, CatBoost, VGG16, and BiLSTM (Table 10).

**Table 10:** Performance of ML and DL models using the FastText technique.

Predictive algorithm	Classification accuracy	Precision	Recall	F1 measure
Machine learning	–	–	–	–
1 Logistic regression	0.86	0.82	0.85	0.83
2 Random forest	0.87	0.84	0.86	0.85
3 Extreme hradient boosting	0.87	0.85	0.87	0.85
4 Decision tree	0.80	0.79	0.80	0.80
5 Support vector machine	0.86	0.83	0.86	0.84
6 CatBoost	0.87	0.83	0.87	0.85
7 AdaBoost	0.85	0.82	0.85	0.83
8 LightGBM	0.86	0.83	0.86	0.85
Deep learning	–	–	–	–
9 VGG16	0.85	0.82	0.85	0.83
10 ResNet	0.84	0.81	0.84	0.83
11 BiLSTM	0.84	0.81	0.84	0.82

The performance comparison between deep learning and machine learning with FastText models demonstrates that XGBoost, Random forest, and CatBoost outperform, with accuracies of 0.87%. XGBoost, in particular, shows strong performance with a precision of 0.85, a recall of 0.87, and an F1 score of 0.85. In comparison, the decision tree performs poorly, with an F1 score of 0.80 and an accuracy of 0.80. Deep learning models such as VGG16 achieved competitive results, with an accuracy of 0.85, while ResNet and BiLSTM each achieved an accuracy of 0.84 (Table 11).

**Table 11:** Performance of ML and DL models using the GloVe technique.

Predictive Algorithm	Classification accuracy	Precision	Recall	F1 measure
Machine learning	–	–	–	–
1 Logistic regression	0.85	0.82	0.85	0.83
2 Random forest	0.86	0.82	0.86	0.84
3 Extreme gradient boosting	0.87	0.83	0.86	0.85
4 Decision tree	0.76	0.75	0.76	0.76
5 Support vector machine	0.87	0.83	0.86	0.85
6 CatBoost	0.87	0.83	0.87	0.85
7 AdaBoost	0.82	0.80	0.82	0.80
8 LightGBM	0.86	0.83	0.86	0.85
Deep learning	–	–	–	–
9 VGG16	0.87	0.87	0.87	0.87
10 ResNet	0.78	0.77	0.78	0.77
11 BiLSTM	0.86	0.83	0.86	0.84

According to the results, XGBoost, SVM, and CatBoost outperformed the other machine learning models, achieving 87% accuracy and strong precision, recall, and F1 scores (0.83–0.87), demonstrating a solid balance between accurately detecting approvals and rejections. With 85–86% accuracy, LightGBM random forest and logistic regression also performed well; however, AdaBoost and the decision tree performed worse, indicating lower prediction reliability and somewhat lower accuracy. VGG16 outperformed all other models in deep learning and produced reliable, robust predictions for accuracy, precision, recall, and F1 score, with an accuracy of 0.87%. BiLSTM trailed closely with 0.86% accuracy, while ResNet’s poor performance (78% accuracy) indicated lower predictive capacity than the other models. Overall, the outcomes of the best deep learning and machine learning models were competitive (Table 12).

**Table 12:** Performance of ML and DL models using the Indic-BERT technique.

Predictive algorithm	Classification accuracy	Precision	Recall	F1 measure
Machine learning	–	–	–	–
1 Logistic regression	0.66	0.59	0.66	0.62
2 Random forest	0.69	0.63	0.69	0.64
3 Extreme gradient boosting	0.74	0.66	0.73	0.68
4 Decision tree	0.56	0.55	0.56	0.55
5 Support vector machine	0.61	0.56	0.61	0.55
6 CatBoost	0.71	0.65	0.71	0.66
7 AdaBoost	0.65	0.58	0.65	0.61
8 LightGBM	0.73	0.66	0.73	0.68
Deep learning	–	–	–	–
9 VGG16	0.61	0.55	0.61	0.58
10 ResNet	0.54	0.29	0.54	0.37
11 BiLSTM	0.56	0.49	0.56	0.52

The findings indicate that Extreme Gradient Boosting was the top-performing machine learning model (74% accuracy) and LightGBM (73%), followed closely by CatBoost (71%). These models demonstrated balanced precision, recall, and F1 scores, indicating consistent predictions. Random forest and logistic regression produced acceptable results, while the decision tree and SVM performed worse. Deep learning performance was generally lower, with VGG16 obtaining 61% accuracy and BiLSTM at 56%. ResNet had the lowest overall performance, with a precision of 0.29 and an F1 score of 0.37, suggesting poor predictive ability. Gradient-boosting models (XGBoost, LightGBM, and CatBoost) beat both classical ML and deep learning approaches in this examination.

#### 4.5.1 Total ML model accuracy

**Figure 14:**
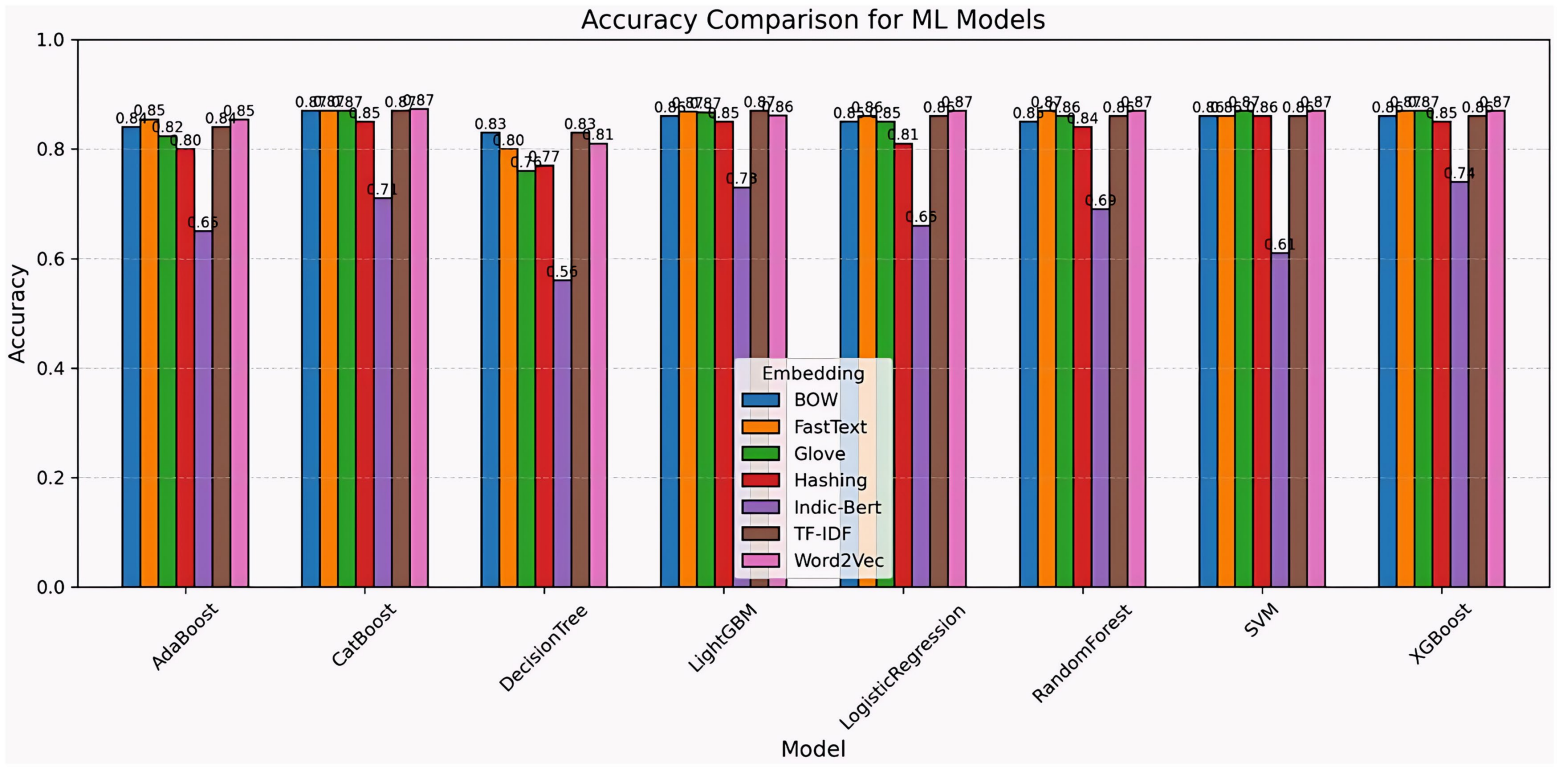
Total ML model accuracy comparison across different embedding techniques.

The accuracy comparison shows that the majority of machine learning models performed well across a range of embedding approaches, including Word2Vec, Indic-BERT, FastText, GloVe, Hashing, BOW (bag-of-words) performance metric, and TF-IDF. The robustness of XGBoost, SVM, CatBoost, random forest, logistic regression, and LightGBM to feature representation techniques is demonstrated by their top accuracies of 0.86–0.87 with little change among embeddings. The accuracy of AdaBoost ranged from 0.65 to 0.85 depending on the embedding, whereas the decision tree’s accuracy decreased significantly, especially with IndicBERT (0.56) and GloVe (0.76). Regardless of the embedding choice, sophisticated ensemble models and gradient boosting techniques often demonstrated stability and improved accuracy, whereas simpler models were more sensitive to the feature representation used (Figure 14).

**Figure 15:**
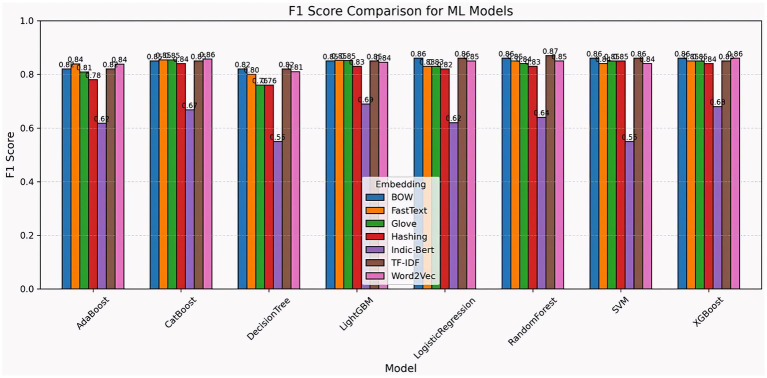
F1 score comparison of ML models across embedding techniques.

The chart compares F1 scores from various machine learning models that use embeddings, including BOW, FastText, GloVe, hashing, Indic-BERT, TF-IDF, and Word2Vec. Logistic regression, random forest, and XGBoost achieved the most consistency, with scores ranging from 0.85 to 0.87. AdaBoost, CatBoost, and LightGBM all perform well, but the decision tree and SVM exhibit more fluctuation, with the decision tree dropping to 0.55 (Indic-BERT) and the SVM dropping below 0.56 in some embeddings (Indic-BERT). BOW, TF-IDF, and FastText all produce superior results, whereas Indic-BERT is less consistent. Overall, ensemble and boosting models perform well across embeddings, making them suitable for text classification applications (Figure 15).

#### 4.5.2 Total Dl model accuracy

**Figure 16:**
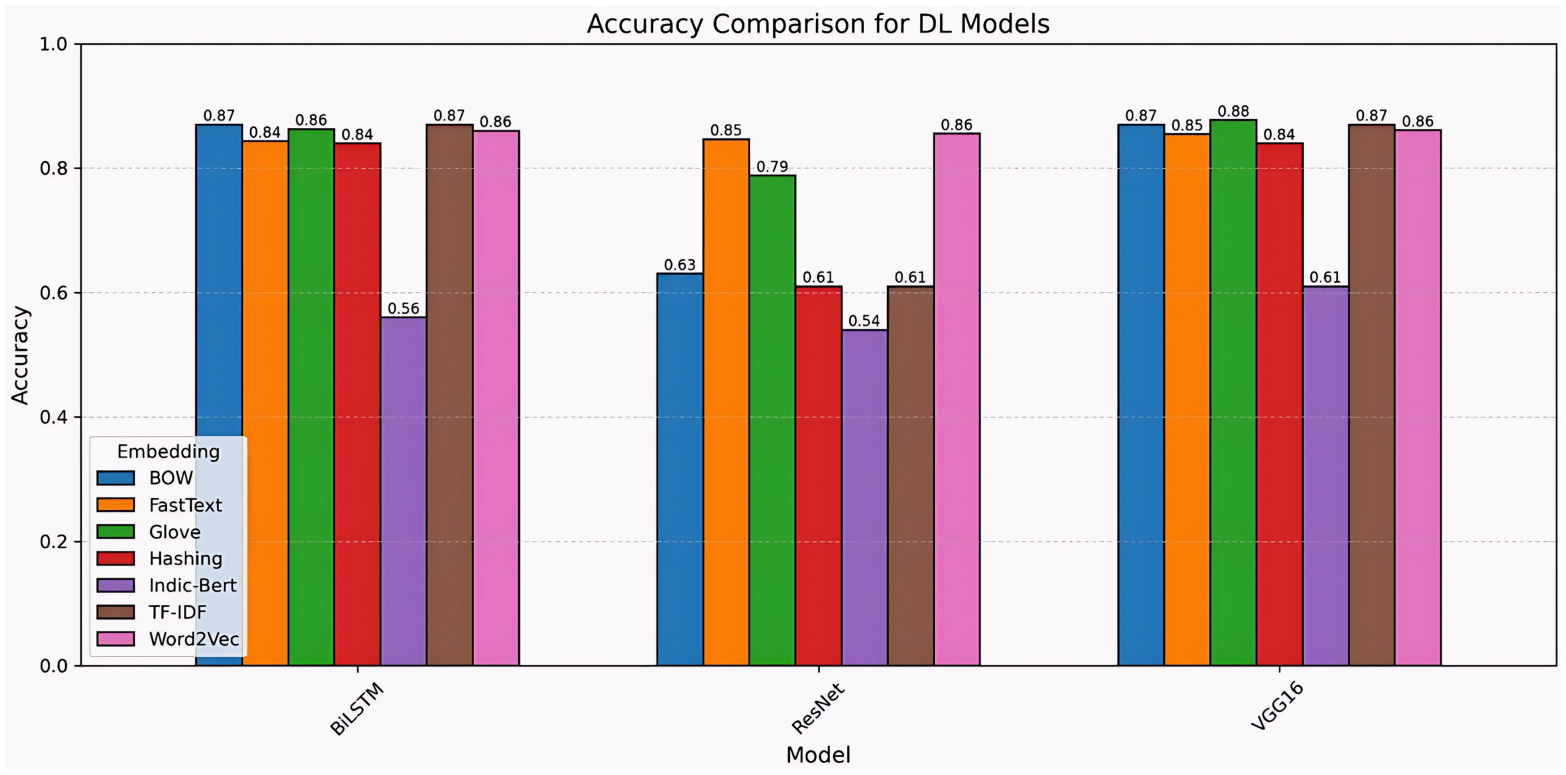
Total DL model accuracy comparison across different embedding techniques.

When comparing the accuracy of deep learning models, VGG16 and BiLSTM consistently outperform ResNet across the majority of embedding approaches. VGG16 achieved the highest accuracy (0.88) with GloVe, followed by BOW, TF-IDF, Word2Vec, and FastText (0.85–0.88), demonstrating strong adaptation across different feature representations. BiLSTM fared well with BOW and TF-IDF, reaching 0.87, but dropped significantly to 0.56 with Indic-BERT, showing sensitivity to certain embeddings. ResNet had the poorest overall performance, with accuracies ranging from 0.54 (Indic-Bert) to 0.86 (Word2Vec), suggesting limited tolerance for alternative embedding types. Overall, VGG16 emerged as the most consistent and accurate model, followed closely by BiLSTM, whereas ResNet struggled with many embedding strategies. Additionally, models such as VGG16, which have stronger feature-extraction capabilities, remain more stable across a range of text representations. These results demonstrate that embedding selection can significantly influence the effectiveness of deep learning (Figure 16).

**Figure 17:**
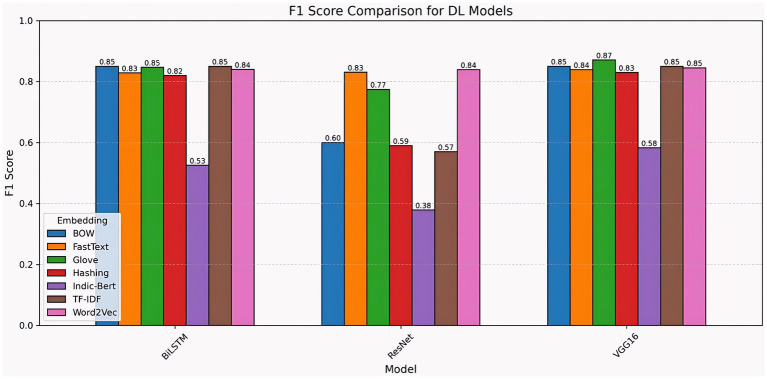
F1 score comparison of ML models across embedding techniques.

The chart compares the F1 scores of deep learning models (BiLSTM, ResNet, and VGG16) utilizing embeddings such as BOW, FastText, GloVe, Hashing, Indic-BERT, TF-IDF, and Word2Vec. VGG16 performs well, notably with GloVe (0.87), and generally scores 0.83–0.85 with other embeddings, with the lowest result for Indic-BERT (0.58). BiLSTM performs well across most embeddings (0.82–0.85), except for Indic-BERT (0.53). ResNet has the greatest variety, with Word2Vec (0.84) performing best and Indic-BERT (0.38) performing worst. Overall, VGG16 and BiLSTM maintain consistent high performance, whereas ResNet’s effectiveness depends largely on the embedding used, underscoring the need to match the proper embedding to the model (Figure 17).

## 5 Discussion

The analysis of users’ reviews reveals substantial variation in public perception across the various money-lending platforms and shows clear patterns in satisfaction and operational efficiency. Overall, most platforms outperform in key financial services such as loan processing, withdrawals, repayment, OTP verification, and document verification, demonstrating customer trust in post-approval processes.

Among the platforms, IndiaMoneyMart has the most favorably rated platform, with consistently high positive satisfaction in core services such as CIBIL handling (100%), OTP (100%), and withdrawals (100%). Users also express positive responses toward remaining features. It suggests exceptional consistency in financial execution. However, this platform receives completely negative feedback on loan rejections (100%) and receives criticism for its poor app interface and weak responsiveness. i2iFunding also performs strongly, excelling in document verification (93.9%) and payment (83.5%), while withdrawals score (84.6%), which indicates reliability in transaction-related functions. Despite this, the platform faces strong criticism for its high rejection rate (88.3%) and weaknesses in app interface, user experience, customer service, and login issues. This indicates reliability in transaction-related functions. Similarly, LendBox also performs well in repayment (82.4%), document verification (80.8%), and the loan process, thereby confirming user trust in its credit handling. Nevertheless, users highlight major drawbacks, including high loan rejection rates (95.9%). LenDenClub and Faircent maintain high positive sentiment for withdrawals (96.8 and 91.8%). Simultaneously, both apps receive positive feedback for document verification (90.9 and 89.9%). However, it still suffers from poor user satisfaction with the application interface, user experience, customer support, and login experience. While CashKumar demonstrates moderate outcomes, it performs well in document verification (87.4%) and withdrawals (83.3%). It receives an average inclusive response (50%) for CIBIL and credit scoring and earns positive feedback on the loan process and OTP verification. Users remain dissatisfied with its loan rejections (88.6%). Finally, 5Paisa achieves the lowest overall satisfaction, despite good performance in CIBIL handling (89.7%), document verification (85.1%), and OTP verification (84.3%), highlighting consistent execution of its core financial tasks. However, it receives negative feedback for loan rejection and the app interface. All platforms received the fewest neutral ratings.

Overall, users are very satisfied with key financial processes, such as loan processing, withdrawals, repayments, and OTP verification, aligning with the Technology Acceptance Model (TAM) and UTAUT framework principles of perceived usefulness and performance expectancy. Platforms such as India Money Mart and i2iFunding excel in these areas. High ratings for loan disbursement, repayment, and withdrawals across most platforms are also consistent with the SERVQUAL dimensions of reliability and responsiveness, which highlight prompt, dependable service as critical drivers of customer satisfaction. Variables such as app interface, login experience, and navigation efficiency are related to perceived ease of usage (TAM) and effort expectancy. Those who have a positive experience with the loan acceptance, withdrawal, and repayment procedures are more likely to continue utilizing these services. Platforms that handle credit ratings with security and transparency align with the trust-risk paradigm. Loan denials and ambiguity erode users’ sense of control and trust, which in turn erodes their confidence that the system will successfully address their financial needs. Login issues and loan denials indicate deficiencies in perceived ease of use and effort expectancy, which are essential obstacles to TAM and UTAUT’s use of technology.

The platform-specific variances in sentiment and topic trends are descriptive contrasts obtained from the review data. There are no inferential statements about the superiority of one platform over another. Some variations across apps were minor and should not be interpreted as statistically significant. The distribution of reviews may be influenced by a variety of contextual factors, such as software maturity, release cycles, and timing of user interactions (Table 13).

**Table 13:** Performance assessment of money lending loan applications.

Topics	5Paisa	Faircent	i2iFunding	LenDen Club	Cash Kumar	Lendbox	India Money Mart
App Interface	62.0% Below Average	87.7% Below Average	86.4% Below Average	72.2% Below Average	78.7% Below Average	82.8% Below Average	71.9% Below Average
Application Experience	52.8% Below Average	68.7% Below Average	73.7% Below Average	62.8% Below Average	65.4% Below Average	87.5% Below Average	63.6% Good
CIBIL and Credit Score	89.7% Good	74.3% Good	84.6% Good	66.7% Good	50% Inclusive	60% Good	100% Good
Customer Service	53.1% Below Average	49.4% Good	73.1% Below Average	60.8% Below Average	81.0% Below Average	90% Below Average	66.7% Good
Document Verification	85.1% Good	87.9% Good	93.9% Good	90.9% Good	87.4% Good	80.8% Good	85.7% Good
Loan Process	63.8% Good	70.5% Good	81.3% Good	88.2% Good	51.4% Good	80.8% Good	97.3% Good
Loan rejection	62.0 % Below Average	79.2% Below Average	88.3% Below Average	70.2% Below Average	88.6% Below Average	95.9% Below Average	100% Below Average
Login issues	48.4% Below Average	70.6% Below Average	59.3% Below Average	67.3% Below Average	56.2% Below Average	86.9% Below Average	52.4% Good
OTP and Verification	84.3% Good	83.1% Good	78.1% Good	89.0% Good	52.0% Good	54.5% Good	100% Good
Repayment and EMI	51.6% Good	62.3% Good	83.5% Good	67.3% Good	70.4% Good	82.4% Good	71.4% Good
Withdrawal	80.2% Good	91.8% Good	84.6% Good	96.8% Good	83.3% Good	62.5% Good	100% Good

Topics were labeled for each platform based on the dominant sentiment in reviews. A topic was rated as good if the proportion of positive reviews exceeded the combined share of neutral and negative reviews, average if neutral reviews accounted for the majority, and below average if negative reviews dominated. When no review data was available for a specific topic on a platform, it was designated as NA (Table 14).

**Table 14:** Overall public perception of digital lending platforms.

SN	App names	Public perception
1	IndiaMoneyMart	Overall best-performing platform, as it received the highest positive ratings across most categories,
2	i2iFunding	Shows strong positive, balanced performance with steady user satisfaction across most categories
3	Lendbox	Shows high satisfaction in usability and service, though loan rejection remains a major drawback.
4	LenDenClub	Good in the loan process and withdrawals, but weak in rejections and login issues.
5	Faircent	Shows mixed sentiment, with a strong app but weak customer service and high loan rejections.
7	CashKumar	It is an average performer with many mixed reviews across categories.
8	5Paisa	Records the lowest overall public satisfaction among the platforms.

Beyond modeling performance, the perception analysis of lending platforms reveals distinct differences in public opinion. Ensemble and gradient boosting classifiers such as XGBoost, SVM, CatBoost, random forest, logistic regression, and LightGBM consistently achieved highest accuracies, ranging from 0.86 to 0.87, indicating strong adaptability to diverse text representations, including BOW, TF-IDF, FastText, Word2Vec, and GloVe. In contrast, simpler models such as AdaBoost and Decision achieved lower accuracies (0.77–0.80), indicating limited generalization across different embedding techniques. Among deep learning approaches, VGG16 consistently performed best, achieving 0.88 accuracy with GloVe embeddings, followed closely by BiLSTM with 0.87 for BOW and TF-IDF. Both models maintained balanced precision and recall, with F1 scores between 0.84 and 0.87, while ResNet showed poor adaptability, dropping to an accuracy of 0.50 under Indic-BERT. These results highlighted that model architecture and embedding compatibility significantly influence predictive performance. According to the findings, the most dependable methods for sentiment and loan classification tasks in digital lending applications are sophisticated ensemble and deep learning techniques, which deliver the most accurate and stable results, with top accuracies of 0.86–0.88 and consistent F1 scores.

As presented in Table 15, the models’ primary results highlight clear patterns in user perception and behavioral patterns in digital lending. Boosting models such as CatBoost, LightGBM, and XGBoost achieved the highest accuracies (0.86–0.87) across all approaches, while the deep learning model VGG16 achieved the overall best accuracy (0.88 with GloVe). These findings demonstrate that hybrid ML-DL frameworks outperform standard models such as decision trees and AdaBoost when processing large-scale unstructured review data. These models’ strong, stable performance reflects their ability to capture emotional tone, contextual semantics, and topic variations in user feedback, which are critical for understanding behavioral constructs such as perceived trust, usefulness, and ease of use, as described in the TAM, UTAUT, and Trust-Risk frameworks. Predictive modeling accuracy translates into clearer behavioral insights. Apps with higher expected sentiment, such as India Money Mart and i2iFunding, correlate with greater user satisfaction and trust, consistent with the performance expectancy and reliability constructs of UTAUT and SERVQUAL, respectively. Platforms such as 5Paisa and Lendbox, which had higher levels of unfavorable sentiment, showed problems with perceived ease of use and service assurance. Overall, the combination of quantitative modeling and behavioral interpretation illustrates how strong ML-DL models not only improve prediction accuracy but also expand theoretical understanding of user adoption, trust, and satisfaction in the digital lending landscape. This combined evidence extends previous research by empirically demonstrating that greater model precision yields more reliable insights into borrower experience and perceived transparency, which are major drivers of long-term FinTech adoption. The inclusion of theory-derived variables enabled the ML models to identify which factors had the greatest influence on user outcomes. SHAP research revealed that utility- and trust-related material elicited positive sentiment, whereas responsiveness and dependability issues elicited negative sentiment. These findings complement TAM and the Trust-Risk paradigm by showing that usefulness and trust enhance positive perceptions, while service quality gaps heighten discontent. The evidence indicates that theory constructs have a meaningful impact on sentiment patterns.

**Table 15:** A concise summary table comparing model performance.

S. no.	Technique	Models	Algorithms	Accuracy	F1Score
1	BOW	ML	CatBoost	0.87	0.85
		DL	VGG16	0.87	0.85
			BiLSTM	0.87	0.85
2	TFIDF	ML	CatBoost	0.87	0.85
			LightGBM	0.87	0.85
		DL	VGG16	0.87	0.85
			BiLSTM	0.87	0.85
3	Hashing	ML	Support Vector Machine	0.86	0.85
		DL	VGG16	0.84	0.83
			BiLSTM	0.84	0.82
4	WordVect	ML	Logistic Regression	0.87	0.85
			Random Forest	0.87	0.85
			Extreme Gradient Boosting	0.87	0.86
			Support Vector Machine	0.87	0.84
			CatBoost	0.87	0.85
		DL	VGG16	0.86	0.84
			BiLSTM	0.86	0.84
5	FastText	ML	Random Forest	0.87	0.85
			Extreme Gradient Boosting	0.87	0.85
			CatBoost	0.87	0.85
		DL	VGG16	0.85	0.83
6	GloVe	ML	Extreme Gradient Boosting	0.87	0.85
			Support Vector Machine	0.87	0.85
			CatBoost	0.87	0.85
		DL	VGG16	0.87	0.87
7	Indic-BERT	ML	Extreme Gradient Boosting	0.74	0.68
		DL	VGG16	0.61	0.58

## 6 Conclusion

This study evaluated 15,408 Google Play Store reviews from seven RBI-approved Indian P2P lending apps (5Paisa, Faircent, i2iFunding, LenDenClub, CashKumar, Lendbox, and IndiaMoneyMart) using an integrated NLP, ML, and DL framework. The combined preprocessing, topic modeling, sentiment analysis, and predictive modeling workflow included data cleaning, LDA topic modeling (11 topics), sentiment analysis using VADER, and a broad suite of classical and deep-learning classifiers. Overall, sentiment was moderately favorable at 55%, with a focus on post-approval activities, including loan processing, withdrawals, EMI repayments, and OTP verification. Negative feedback (40.96%) was related to onboarding, interface, login issues, loan denials, and CIBIL. The comparative findings reveal various strengths and shortcomings among the lending systems. The results demonstrate that robust text categorization depends more on careful selection of models and architectures than on any single embedding technique. In terms of user impressions, the studies show clear differences among platforms. IndiaMoneyMart and i2iFunding receive the highest ratings, indicating consistent satisfaction with core lending services, including loan processing, verification, and withdrawals. Lendbox and LenDenClub both perform well in these areas but face frequent concerns about interface design, login issues, and transparency around rejections. Faircent and CashKumar have received mixed reviews, with strengths in withdrawals and service but issues with support and loan processing.

In contrast, 5Paisa receives the most unfavorable feedback since its powerful CIBIL and verification features are balanced by poor repayment, login, and user experiences. Among the models tested, boosting classifiers (XGBoost, CatBoost, and LightGBM) and the deep learning architecture VGG16 consistently provided the most reliable performance, boosting models’ high accuracies (0.86–0.87), and VGG16 had the study’s best single accuracy (0.88 with GloVe), confirming their suitability for text-based financial applications. India Money Mart is the most well-liked app, with consistently high ratings for CIBIL handling, loan processing, OTP verification, and withdrawals. Finally, 5Paisa has the lowest overall satisfaction: despite great results in CIBIL handling, document verification, and OTP verification, it receives overwhelmingly unfavorable feedback on repayment processes, login functionality, loan denials, and general application use. The importance of the TAM, UTAUT, SERVQUAL, and Trust-Risk frameworks in digital lending contexts was reinforced by empirical evidence of their impact on user sentiment, as evidenced by the incorporation of theory-derived features into ML models. Overall, these findings highlight the need to adopt robust analytical models while addressing usability, transparency, and customer service issues in digital lending ecosystems to increase user trust and satisfaction.

### 6.1 Practical implication

Managerial and policy implications follow immediately. Platforms should prioritize UI/UX redesign, strengthen authentication/login procedures, increase transparency about rejection criteria and credit-score consequences, and invest in proactive customer support for high-friction steps (onboarding, verification, and rejections). Large-scale review mining provides regulators and consumer-protection agencies with a low-latency signal to monitor market behavior and identify recurring harms (e.g., opaque rejections and aggressive collection), in addition to standard supervisory instruments.

### 6.2 Future directions

Future studies can be expanded to include all remaining RBI-approved P2P lending applications, thereby providing a more comprehensive assessment of the Indian digital lending ecosystem. Furthermore Compare RBI registered apps and non registerd apps. They include human-annotated, multilingual corpora, longitudinal tracking, fairness and interpretability audits of top models, and correlations between sentiment and operational KPIs such as approval times, defaults, and repayment outcomes. Combining review signals and structured telemetry can improve early warning and product-quality analytics in digital lending. The dataset can also be expanded to include evaluations from different platforms (iOS, web portals, social media, and consumer forums) to provide a more complete picture of user perceptions. Multilingual reviews and complex preparation approaches can help capture regional language nuances and subtle expressions that are often lost in ASCII-based filtering. In addition.

## Data Availability

The datasets presented in this study can be found in online repositories. The names of the repository/repositories and accession number(s) can be found in the article/supplementary material.
